# One Health and Cattle Genetic Resources: Mining More than 500 Cattle Genomes to Identify Variants in Candidate Genes Potentially Affecting Coronavirus Infections

**DOI:** 10.3390/ani12070838

**Published:** 2022-03-26

**Authors:** Samuele Bovo, Giuseppina Schiavo, Luca Fontanesi

**Affiliations:** Department of Agricultural and Food Sciences, University of Bologna, Viale Giuseppe Fanin 46, 40127 Bologna, Italy; samuele.bovo@unibo.it (S.B.); giuseppina.schiavo2@unibo.it (G.S.)

**Keywords:** *Bos taurus*, breed, genetic variability, infectious disease, livestock, mutation, SARS-CoV-2, single amino acid polymorphism, zoonosis

## Abstract

**Simple Summary:**

The conservation and exploitation of cattle genetic resources for selection and breeding purposes are important for the definition of sustainable livestock production sectors. One Health approaches should be integrated into these activities to reduce the risk posed by many zoonoses. Coronaviruses are emerging as important zoonotic agents, with the potential to easily cross species barriers, as also recently demonstrated by the COVID-19 pandemic derived by SARS-CoV-2. Genetic resistance to coronavirus infections can be determined by variants of the host (animal) genome segregating within species. In this study, we mined the genome of more than 500 cattle to identify variants that could be involved so as to define different levels of susceptibility and/or resistance to coronavirus diseases in this important livestock species. Using comparative analyses across species, we identified several single amino acid polymorphisms that might alter the function of key proteins involved in the basic biological mechanisms underlying the infection processes in cattle. This study provided new elements to consider genetic variability of the host (cattle) as a potential risk factor to be considered in One Health perspectives.

**Abstract:**

Epidemiological and biological characteristics of coronaviruses and their ability to cross species barriers are a matter of increasing concerns for these zoonotic agents. To prevent their spread, One Health approaches should be designed to include the host (animal) genome variability as a potential risk factor that might confer genetic resistance or susceptibility to coronavirus infections. At present, there is no example that considers cattle genetic resources for this purpose. In this study, we investigated the variability of six genes (*ACE2*, *ANPEP*, *CEACAM1* and *DPP4* encoding for host receptors of coronaviruses; *FURIN* and *TMPRSS2* encoding for host proteases involved in coronavirus infection) by mining whole genome sequencing datasets from more than 500 cattle of 34 *Bos taurus* breeds and three related species. We identified a total of 180 protein variants (44 already known from the ARS-UCD1.2 reference genome). Some of them determine altered protein functions or the virus–host interaction and the related virus entry processes. The results obtained in this study constitute a first step towards the definition of a One Health strategy that includes cattle genetic resources as reservoirs of host gene variability useful to design conservation and selection programs to increase resistance to coronavirus diseases.

## 1. Introduction

One Health strategies, defined as the collaborative efforts of multiple disciplines working locally, nationally, and globally, to achieve optimal health for people, animals, and our environment [1], are required to face the burden caused by emerging diseases. It is estimated that about 60% of emerging infectious diseases globally reported are zoonoses and ~72% of them derive from pathogens of wildlife origin [2]. The pandemic coronavirus disease 2019 (COVID-19) can be considered as one of the latest examples of zoonosis [3,4,5,6,7,8,9] according to the probable bat or pangolin origin [9,10,11]. Zoonotic diseases caused by several coronaviruses (CoV) are not entirely new as these viruses constantly and silently circulate or emerge and re-emerge [12,13,14,15,16,17]. Furthermore, COVID-19 is just the third deadly coronavirus pandemic to emerged in the past two decades, preceded by the outbreak of the severe acute respiratory syndrome coronavirus (SARS-CoV-1; 2002–2003; of putative bat origin) and the Middle East respiratory syndrome coronavirus (MERS-CoV; 2012-present; of putative bat origin [17,18,19]).

Coronaviruses that specifically infect livestock species emerged and spread over the last century causing epidemics that had a negative economic impact on agriculture and livestock industries. These include the infectious bronchitis virus (IBV; 1930s) identified in domestic fowls, the transmissible gastroenteritis virus (TGEV; 1946), porcine respiratory coronavirus (PRCoV) and porcine epidemic diarrhea virus (PEDC; 1971) identified in the domestic pigs [20]. Some CoV have been shown to be multi-host pathogens as they require an intermediate host before being able to infect humans (e.g., cattle for HCoV-OC43, alpacas for HCoV-229E, palm civets for SARS-CoV and dromedary camels for MERS-CoV). At present, Bovine coronavirus (BCoV) is the only known CoV mainly infecting the cattle (*Bos taurus*), with several isolates which have high morbidity but low mortality [21,22,23]. The following elements underline call for One Health strategies in the face of these infecting agents: (i) the biological characteristics of most CoV and their ability to cross species barriers, (ii) the high mutation rate of the CoVs, which can determine quite a large host spectrum for every CoV) and (iii) the high potential negative impacts of many CoVs.

The infection mechanisms of CoVs have been well defined. Viruses enter the host cell after the fusion of the viral envelope with the cell membrane that is triggered by the interaction and binding of the viral spike (S) glycoprotein with the host receptor on the cell surface [24]. Several host receptors are involved in this process and their specificity in binding a target CoV represents the first critical point for viral infection. Four main host protein receptors for CoV [25], encoded by the corresponding host genes, are known: (i) Angiotensin-converting enzyme 2 (ACE2), receptor for HCoV-NL63, SARS-CoV and SARS-CoV-2, (ii) Aminopeptidase-N (ANPEP or APN), receptor for TGEV, PRCoV and HCoV-229E, (iii) Carcinoembryonic antigen-related cell adhesion molecule 1 (CEACAM1), receptor for MHV and (iv) Dipeptidyl peptidase-4 (DPP4), receptor for MERS-CoV and bat coronavirus HKU4. As a result of the virus–host interaction, the host proteases Transmembrane Serine Protease 2 (TMPRSS2) and FURIN cleave the spike glycoprotein and promote the fusion of viral and cellular membranes and complete virus entry [25]. Recent studies have demonstrated that neuropilin-1 (NRP1) can be considered a post-proteolysis host cell receptor for the SARS-CoV-2 as NRP1 binds the furin-cleaved SARS-CoV-2 S1 protein [26,27,28].

Genetic diversity affects the coronavirus-related host genes and thus the encoded receptors or enzymes can alter the susceptibility of the host to coronavirus infection capability and disease progression as already demonstrated for SARS-CoV-2 in different human cohorts [29,30,31,32,33]. In most livestock species, however, it is not yet known if and how genetic variability that might be present in key host genes could affect individual animal sensitivity to CoV infections and, eventually, the spectrum of animal hosts. As a first attempt in this direction, we recently mined the genome of many pigs of different breeds and populations to identify natural variants in key host genes that might affect susceptibility of pigs to CoV infections [34]. We identified a few thousand polymorphisms in the targeted genes and inferred, with an in silico analysis, the potential impact that this might have on protein structure or activity to advance, facilitate or prevent CoV infection of the host. This approach made it possible to propose a novel One Health concept that considers genetic diversity within species as a potential assessment risk against CoV infections that would be important in the conservation strategy of livestock genetic resources [34]. It is therefore important to expand this investigation to other livestock species and further refine the concept based on the predictive ability and extension of the genome-mining approaches. Cattle are one of the most relevant livestock species that includes many different breeds that constitute unique genetic resources.

In this study, we mined more than 500 cattle genomes from 34 different populations or breeds raised in different countries to identify variants in six coronavirus-related host genes (*ACE2*, *ANPEP*, *CEACAM*, *DPP4*, *FURIN* and *TMPRSS2*) that encode for receptors or protease for priming the infection of CoV. Using a comparative genome approach, the identified variants were then analyzed to infer their relevance in terms of conferring potential differences in susceptibility or resistance to CoV infections. The results could be useful (i) to establish a risk evaluation system in a “One Health” approach, including information on the genetic diversity of cattle populations, similarly to what we already proposed in pigs [34]; and (ii) to identify natural genetic variability in cattle that could be considered in genomic selection strategies to increase genetic resistance against emerging and re-emerging CoV diseases in this species.

## 2. Materials and Methods

### 2.1. Animals and Re-Sequencing Datasets

Sequencing data produced from whole genome re-sequencing of cattle DNA samples (including wild populations and other phylogenetically close species) were retrieved from the European Nucleotide Archive (ENA; https://www.ebi.ac.uk/ena/; accessed on 14 September 2020) [35]. As a first step, ENA metadata related to the genus *Bos* (taxid: 9903) were downloaded and filtered. We retained only those samples with the following tags: library_source = GENOMIC, library_layout = PAIRED and library_strategy = WGS. As the number of sequenced nucleotides was available, we filtered out samples having an estimated sequencing depth lower than 5×.

This resulted in a dataset including 503 sequenced genomes from 34 populations of the *Bos* genus, including *B. taurus* (n. 498 African and non-African/cosmopolitan anicattle), *B. indicus* (n. 1), *B. javanicus* (n. 1) and *B. grunniens* (n. 3). Animals of the *B. taurus* species included the following breeds: Angus (n. 28), Blonde d’Aquitaine (n. 1), Brown Swiss (n. 8), Charolais (n. 29), Chianina (n. 3), Gelbvieh (n. 25), Hereford (n. 33), Holstein (n. 121), Jersey (n. 14), Limousin (n. 6), Maine Anjou (n. 12), Original Braunvieh (n. 10), Piedmontese (n. 8), Red Angus (n. 10), Romagnola (n. 7), Salers (n. 8), Shorthorn (n. 13), Simmental (n. 42), Tyrolean Grey (n. 8), Afar (n. 9), Arsi (n. 10), Barka (n. 8), Butana (n. 19), Ethiopian Boran (n. 10), Fogera (n. 9), Goffa (n. 10), Horro (n. 11), Kenana (n. 4), Mursi (n. 10), N’Dama (n. 3) and Sheko (n. 9).

Sequencing data (fastq files) were locally downloaded via the Aspera *ascp* command line client (https://www.ibm.com/products/aspera; accessed on 14 September 2020). Information on all analyzed datasets is provided in Table S1.

### 2.2. Sequence Alignment and Variant Detection

Reads were aligned on the reference cattle genome ARS-UCD1.2 using BWA-MEM v.0.7.17 and Samtools v.1.10 [36,37]. Next, a deduplication step was carried out with Picard v.2.1.1 (https://broadinstitute.github.io/picard/; accessed on 14 September 2020). A summary of whole genome sequencing data statistics is reported in Table 1 and Table S1. Variants were identified in six candidate genes involved in CoV infections (*ACE2*, *ANPEP*, *CEACAM1*, *DPP4*, *FURIN* and *TMPRSS2*; Table S2) via the GATK v.4.1.8.1 [38] HaplotypeCaller algorithm. A joint variant calling approach with GATK4 v.4.1.8.1 GenotypeGVCFs was applied. Only biallelic variants covered by at least three reads in each analyzed genome were retained. The transition-to-transversion ratio (Ts/Tv) was used as a quality indicator [39]. Variants were annotated using the Variant Effect Predictor (VEP) v.95.0 [40] by predicting their impact on the protein function with SIFT v.5.2.2 [41]. Variants that affected the protein-coding regions (i.e., missense, frameshift and stop gain/loss variants), hereafter denoted also as single amino acid polymorphism (SAP), were retained and manually checked. Based on the identified genotypes, allelic frequencies were estimated within each population. Allele frequencies were used as an input for the Principal Component Analyses (PCA).

In additionto the derived mined datasets, the SAP affecting the six analyzed genes were downloaded from Ensembl release 104 [42], presented as information annotated against the reference cattle genome ARS-UCD1.2.

Bioinformatic pipelines were developed in Python v.2.7.12 and in R v.3.4.4 [43].

### 2.3. Comparative Analysis between Cattle and Human Protein Sequences of Candidate Genes

The sequence identity was obtained via alignments carried out with Clustal Omega [44], as implemented in UniProt [45]. Details about genes, transcripts and protein accession numbers used in this analysis are reported in Table S2. The identification of protein residues functionally relevant for CoV infections in humans and animals (SARS, MERS and the novel COVID-19) was carried out via a survey of the literature mainly describing their 3D structural architecture and functional mechanisms. Our attention was focused on those residues (key residues) that have a functional role in the biological activity of the six selected proteins (virus–host interaction, protein processing and signaling), including active sites, substrate sites, ion-binding sites, residues in interaction patches and glycosylation sites. Details about these sites and the surveyed literature are provided in Tables S4–S9. Sequence alignments were evaluated for the identification of conserved and non-conserved functional residues between the human and cattle proteins.

### 2.4. Overview of the Bioinformatic Pipelines

**Figure 1:**
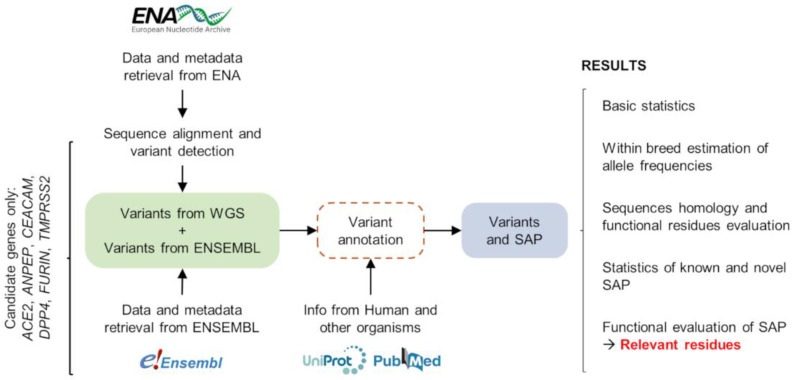
Flowchart of the bioinformatic analyses implemented for the identification of relevant variants in cattle genome datasets.

A flowchart of the bioinformatic analyses adopted in this study is presented in Figure 1. Briefly, sequencing data were downloaded from a public database and processed. DNA variants were identified in the candidate genes, annotated and residues relevant for the protein function and virus–host interaction were evaluated across species. The results of each step are detailed in the following paragraphs.

## 3. Results

### 3.1. Sequencing Results and Variability Detected in Candidate Genes

A total of 498 publicly available whole genome re-sequencing datasets of *B. taurus*, derived from nine sequencing projects, were used in this study, including 12 African and 19 non-African populations. On average, each population was represented by 14 animals (min = 1 for Blonde d’Aquitaine; max = 121 for Holstein). We added a few samples of other cattle populations and phylogenetically close species including *B. t. indicus* (n. 1), *B. javanicus* (n. 1) and *B. grunnies* (n. 3).

Around 187 billion reads accounting for more than 13 Terabytes of raw data were processed. Each dataset had an average ± standard deviation (s.d.) breadth of sequencing of about 99 ± 2%. The depth of sequencing was on average equal to 16 ± 6X and, stratified by population, ranged from 10X to 32X for Blonde d’Aquitaine (n. 1 sample) and Shorthorn (n. 13 samples), respectively. Detailed statistics for these sequencing parameters are reported in Table S1.

A total of 6565 variants (5942 SNPs and 623 indels) were identified in the six candidate genes. The Ts/Tv ratio was equal to 2.3, a value that is consistent with what has been observed in other mammalian organisms (e.g., [34]).

**Figure 2:**
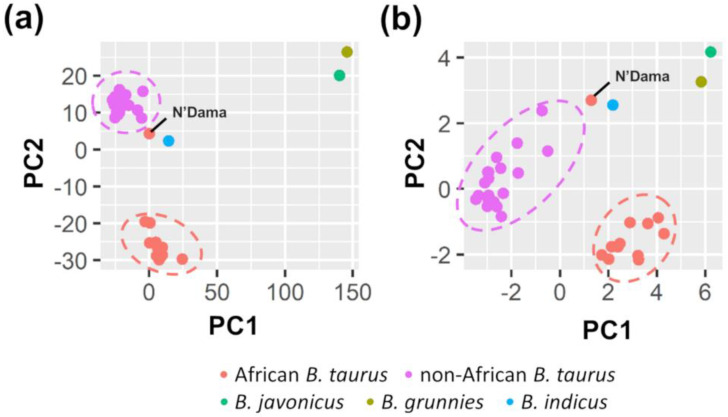
Principal Component analysis of the 34 *Bos* populations based on the allele frequencies of variants identified in the six coronavirus-related genes. Principal components (PC) 1 and 2 are shown. (**a**) The whole set of identified variants (n. 6565) was used; (**b**) Only variants affecting the protein and present in at least two *B. taurus* populations were considered (n. 82).

For each variant, we computed the frequency of the alternative allele in the 35 populations. Based on these data, the PCA analysis evidenced two main clusters corresponding to African and non-African cattle (Figure 2a). The results were in line with a previous study that analyzed the whole genetic variability in these populations [46]. As expected, the *B. indicus* sample was close to the *B. taurus* sample, as it is nowadays considered a subspecies (*B. t. indicus*). However, we preferred to treat it separately. *Bos javanicus* and *B. grunniens* clustered apart. Moreover, the African N’Dama breed was separated from the other African breeds as also observed by [46]. N’Dama was genetically closer to the non-African breeds and *B. t. indicus*. As several variants (n. 1995; 30%) were identified in only the *B. t. indicus*, *B. javanicus* and *B. grunniens* species, further analyses were also carried out in which those variants were excluded (Figure S1). However, the results did not change.

Approximately 93% of the variants detected in at least one *B. taurus* breed were SNPs. The remaining 7% were indels. Quite a large fraction of these variants (74%) was already known and already have a dbSNP Reference SNP (rs) number. On average, each gene had 67 ± 17% known variants. The *FURIN* gene had the lowest number of known variants (31%). Overall, the largest fraction of variants (~90%) was within introns. Each gene had 80 ± 20% intronic variants. The *FURIN* gene was an outlier for this parameter as it was characterized by 64% of variants located in the untranslated regions (UTRs) + exons, followed by *ANPEP* (26%) and *CEACAM1* (16%) genes.

### 3.2. Protein Variants Affecting Candidate Genes

**Figure 3:**
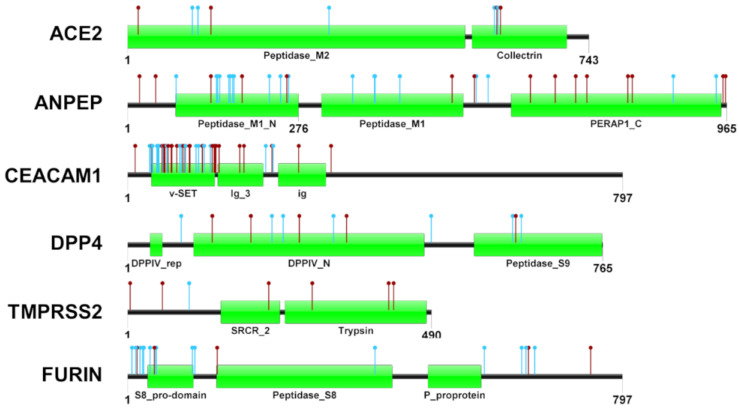
Variants affecting the protein coding sequence of the six coronavirus-related genes. Red dots indicate the variants retrieved from Ensembl database, whereas light blue dots indicate variants identified in the whole genome sequencing datasets. Protein domains and their coordinates are based on the Pfam database (https://pfam.xfam.org/; accessed on 14 September 2020) considering the protein identifiers provided in Table S2.

Coronavirus receptors and proteinases have been extensively studied in humans (see the full set of references cited in the Supplementary Materials), whereas little information is available with regard to farm animals. To further characterize and infer a potential functional effects of protein variations, we performed comparison between human and cattle protein sequences followed by mapping of variants identified via WGS in the present study. Figure 3 shows the architecture of the analyzed and the set of variants identified in cattle, which were derived by combining the different datasets explored in this study (WGS data and the Ensembl database).

The sequence homology between the cattle and human (or mouse) protein sequences (Table S2) was equal to 74% for ACE2, 78% for ANPEP, 48% for CEACAM1, 89% for DPP4, 78% for TMPRSS2 and 95% for FURIN.

By mining the UniprotKB database and the literature survey, a total of 82 (ACE2), 28 (ANPEP), 38 (CEACAM), 24 (DPP4), 17 (FURIN) and 8 (TMPRSS2) residues essential for the protein functional activity (Tables S4–S9) were identified. These key residues presented a variable degree of conservation between cattle and the other organisms (mainly human), with a total of 47/82 (57%), 19/28 (68%), 10/38 (26%), 26/30 (87%), 8/8 (100%) and 17/17 (100%) identical key residues (Tables S4–S7), respectively.

The Ensembl database found a total of 708 protein residues of the six candidate genes that were affected by variability. These residues represented 8–30% of the whole protein sequences. The number of SAP did not correlate with the length of the protein sequences. It is interesting to note that only few (n. 62/708) were detected in the investigated WGS datasets. Figure 3 shows the position of all variants deposited in Ensembl and the newly discovered variants in the mined WGS datasets.

The sequencing data returned a total of 180 different variants (for the whole *Bos* genus) affecting 165 residues of the coding sequence of the candidate genes. A total of 136 variants of the 180 discovered (~75%) were present in at least one *B. taurus* breed, whereas the remaining variants characterized the other *Bos* species. Overall, about half of the discovered variants were novel (72/136 = 0.53), whereas the other half presented a dbSNP Reference SNP (rs) number. In addition, 34/136 (25%) were also detected in the other related *Bos* species.

**Figure 4:**
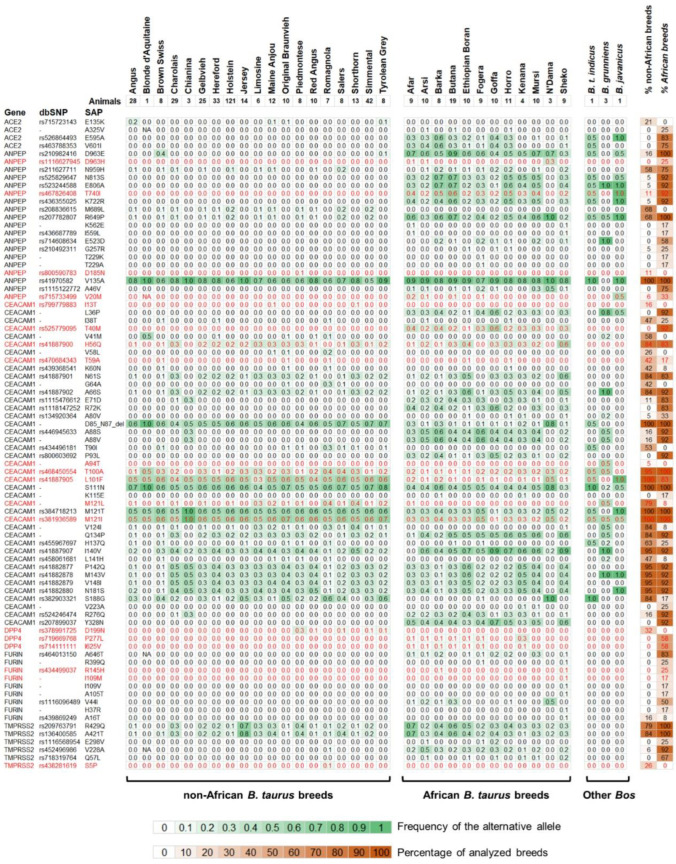
Allele frequency (AF) of the alternative allele of the protein variants (SAP) identified from the sequencing data. Only variants affecting the protein and detected in at least two *B. taurus* populations are reported (n. 82 variants). Cattle populations are divided in non-African *B. taurus*, African *B. taurus* and other *Bos* species. SAP predicted as deleterious for the protein function by SIFT are evidenced in red. Detailed information is provided in Table S3.

The PCA based on the full set of 136 variants was in agreement with our previous finding relying on the whole variation set (UTRs + introns + exons) as (i) the two main clusters of African and non-African cattle populations were preserved; (ii) *B. t. indicus*, *B. javanicus* and *B. grunniens* species clustered apart; and (iii) N’Dama breed was not closer to the African cluster. This position was also maintained when we excluded private variants (Figure 2b). A total of 82 variants out of 136 (60%) were segregated in at least two *B. taurus* populations. The allele frequency is shown in Figure 4 and reported in Table S3. The remaining 54 variants were exclusive to a given breed. Their allele frequency is presented in Table S3.

### 3.3. Functional Evaluation of Protein Variants

The impact at the protein functional level was evaluated for the whole set of variants discovered in cattle breeds from WGS. We initially evaluated non private variants present in at least two *B. taurus* populations (n. 82 variants).

Firstly, ACE2 had four missense variants predicted, as tolerated by SIFT. The single amino-acid polymorphism p.E595A located in a protein region interacting with the ADAM17 sheddase was observed in more than 80% of the African breeds.

Variants identified in the *ANPEP* gene included 19 missense mutations, four of which were predicted to be deleterious by SIFT. In particular, two putative deleterious variants (p.V20M and p.T40I) were present in more than 90% of the African breeds. Overall, eight of nineteen variants were present in more than 75% of the African breed. Variant p.V135A was present in all African and non-African populations at high frequency. Variants p.K722R, p.T740I, p.E806A and p.N813S were located within the region responsible for the interaction between the porcine ANPEP and the viral TGEV and PRCV glycoproteins [47].

Additionally, CEACAM1 had 40 missense variants and one in-frame deletion. Nine missense variants were predicted by SIFT as deleterious for the protein function and two of them (p.L101F and p.M121I) generally had a high frequency in several populations. Overall, 24 and 18 variants were present in more than 75% of non-African and African populations, respectively. The in-frame deletion p.D85_N87_del, characterizing all breeds, had a generally higher allele frequency in non-African populations compared to the African cattle. This mutation affects one of the 18 residues involved in the interaction with CoV. Moreover, we identified other five variants (p.G64A, p.A88S, p.A88V, p.T90I, and p.P93L) that affect the virus–host interaction sites.

In addition, DPP4 was characterized by three missense variants predicted as deleterious by SIFT. Overall, these variants had a low allele frequency (<30%). Variants p.P277L and p.I625V were identified in 58% of African breeds, whereas p.D199N was present in 32% of non-African populations.

FURIN counted nine missense variants. Two of them were predicted as deleterious by SIFT but were present with a low allele frequency (<10%) in a few (n. 3) African breeds. In general, variants were discovered mainly in non-African populations.

Furthermore, TMPRSS2 had six missense variants. Only one variant was predicted to be deleterious by SIFT and it was found mainly in non-African breeds at a low frequency.

In the evaluation of these six coronavirus-related genes, we did not find any variants affecting relevel functional sites (e.g. active sites, substrate sites, ion-binding sites, residues in interaction patches and glycosylation sites).

*B. grunniens* and *B. javanicus* counted a total of 20 (24%) and 16 (19%) variants, respectively.

With regard to the 54 remaining private variants, most of them had a lower frequency of the alternative alleles. Variants ACE2 p.M18I in Jersey, DPP4 p.S332F in Shorthorn and DPP4 p.N137K in N’Dama had allele frequencies >25%. About ⅔ of private variants derived mainly from African populations.

## 4. Discussion

Defining a comprehensive list of factors contributing to the emergence and transmission of a disease from animal to human (and vice versa) is a key step for the development of risk assessments and surveillance plans, especially in a One Health perspective [48]. In this context, animal selection and breeding programs, with the objective of the improvement of the genetic resistance of livestock populations to infection diseases, can be considered key elements for a sustainable animal production sector. This is of course related to the fact that (i) animals resistant to infection diseases need less antimicrobial or drug treatments, with a contribution to reduce antimicrobial resistance, drug residues in animal products and reduction of veterinary costs, (ii) healthy animals are more productive, with a positive impact on farmers’ income and (iii) animal welfare is pursued when infection diseases do not hit animals. Another factor that integrates these elements follows One Health concepts: if animals are genetically more resistant or less susceptible to diseases, on one hand there is a reduced risk to transmit zoonotic infections to humans and on the other hand potential pathogens of human origin transmitted to the animals might have a limited negative impact in the livestock sector. A few examples that included viruses infecting livestock and that followed the animal–human and human–animal transmission routes, have been already reported (e.g., [49]). In addition, from another “One Health” perspective, conservation strategies of animal genetic resources should also evaluate the risk derived by the fixation or high frequency in a population of alleles associated with susceptibility (that are a matter of concern) or resistance (that are evaluated positively) to diseases of potential zoonotic relevance.

In practice, for several reasons, it is very difficult to dissect the genetic components and identify genetic variants associated with or determining susceptibility/resistance to infectious diseases in livestock species [50]. For example, (i) disease resistance as a phenotype is difficult to measure and some proxies or alternative traits should be considered, (ii) G×E interactions largely affect disease susceptibility/resistance, (iii) disease resistance is a complex trait usually determined by many genes with small effects and (iv) the genetic architecture of disease resistance may vary according to the type of pathogen and the host. In addition, large and expensive experimental designs should be used to obtain meaningful information from a genetic point of view. A few studies, for example, were designed to dissect the cattle genome for the identification of QTL affecting CoV related diseases [51,52,53,54]. Despite the extensive efforts that have been carried out, limited results that could be useful in selection programs have been obtained. Therefore, alternative strategies or approaches should be explored.

High throughput sequencing has opened new opportunities for large scale analyses of the animal (host) genome of many individuals. Mining large sequencing datasets can help to identify natural variants with potential effects on susceptibility/resistance to infective agents. Following this, comparative genome analyses across species can help to further strengthen the identification of candidate variants when coupled with predicted functional effects. This combination of approaches takes advantage of what is known from basic biology concepts, results determined from in vitro or in vivo models or determined in one species and then transferred in another species. Genome mining and functional inference can be considered a part of the more articulated systems biology approach [55]. Even if caution should be used for the interpretation of results derived by this process, and validation studies should be set up, in this way it is possible to accelerate the identification of variants that might be involved in disease susceptibility or resistance. This is particularly true if applied to CoV-derived diseases, as many results have been obtained in this field over the last few years, stimulated by the COVID-19 pandemic.

We already proposed this bottom-up approach to analyze the pig genome and detect variants implicated in the variability of coronavirus infection resistance [34]. In the current study, we transferred and expanded (by analyzing a larger number of candidate genes and WGS datasets) a similar approach to cattle. Whole genome resequencing datasets produced from 503 cattle belonging to 34 groups (breeds or species) were mined to identify genetic variants in genes encoding for protein receptors (*ACE2*, *ANPEP*, *CEACAM* and *DPP4*) and proteases (*FURIN* and *TMPRSS2*) involved in the mechanisms of CoV infection [24,25]. We identified variants affecting the protein sequences that were evaluated, in a comparative analysis, with what is known about the corresponding human (and other CoV hosts) gene products. Considering that proteins are characterized by fundamental residues for their functional activity (e.g., residues involved in the virus–host interaction or residues forming the active sites), comparative analyses focused mainly on single amino acid polymorphisms (SAP). However, as in the present comparative analysis, we did not consider variants of flanking regions and of introns (the most frequent types of variants in our mining) with potential regulatory roles; thus, we may have only partially explored the variability of the selected genes. It is possible that regulatory variants in these regions alter the gene expression of a given receptor or effector protein and, in turn, affect the basic mechanisms that lead to CoV infections in the host. Transcriptomic analyses and the use of other omic approaches could complement genomic datasets even if their exploitation in this context is quite challenging and might require integration into a systems biology strategy.

Variants identified in these six genes were initially used to study the population structures. What emerged was a division in clusters of the investigated cattle populations that clearly produced three groups: African breeds, non-African breeds and other animals of the *Bos* genus. Despite the fact that variability in only six genes was used in this cluster analysis, the results confirmed the information obtained by analyzing whole-genome variability [45]. Therefore, these six candidate genes carry ancestral signatures that are consistent with the evolutionary and domestication processes of the cattle.

The identified variants were enriched with polymorphic sites already described in the *Bos taurus* genome. Among the 180 SAP that we identified, a total of 66 SAP were already known and included in the list of 708 SAP that were retrieved from the annotation available in the ARS-UCD1.2. reference genome and Ensembl database. Therefore, the SAP obtained from our mining of more than 500 cattle genomes and from the Ensembl database matched only about 10% of these types of variants. This low level of matching could be explained by the fact that (i) part of the 708 SAP are due to rare mutations that we did not detect even in our large survey of sequenced genomes and (ii) the annotated variants in the Ensembl database, derived by other non-quality checked sources (e.g., dbSNP database), may have contained sequencing errors. To support these hypotheses, we checked the data retrieved from Ensembl database and counted only a total of 70 out of 708 SAP presenting multiple observations. It turned out that 44 out of these 70 variants (63%) were also in our WGS datasets.

SAP-affecting protein receptors included some interesting functional residues (ACE2 p.A595; CEACAM p.G64A, p.A88S, p.A88V, p.T90I, p.P93L and ANPEP p.K722R, p.T740I, p.E806A and p.N813S) as they might change the potential association between the CoV spike protein and the host receptor. Analyses of protease-coding genes (*FURIN* and *TMPRSS2*) did not evidence any relevant variants affecting functional sites even if a few SAP were predicted by SIFT as deleterious for the protein function. These variants could be strong candidates for the modification of the response or sensitivity of the host to CoV infection. It could be important to further evaluate the function of these variants with more advanced 3-D modelling and protein-interaction studies. In vitro analyses would then be needed to validate these in silico predictions.

Several studies have attempted to predict the host range of SARS-CoV-2 by comparative and structural analysis of its specific receptor (ACE2) in vertebrates, including in cattle and other Bovidae species [33,56,57,58,59,60]. The protein sequence of the cattle ACE2 receptor might potentially be partially compatible in the binding of the S protein. That means that cattle could be potentially susceptible to SARS-CoV-2 infection. However, the studies of experimentally infected cattle with SARS-CoV-2 did not confirm that all animals of this species could be highly susceptible to the virus, and the possibility of SARS-CoV-2 to infect or multiply in the host varied among individual animals [61,62]. All these studies, however, did not consider any within-species variability that indeed might be quite common and that could be associated with different susceptibility to the infection, at least in humans [31]. In our WGS mining, we only identified an SAP in the cattle ACE gene that is predicted to affect the function of the encoded receptor. It would be interesting to evaluate if this variability might be responsible for the observed differences in SARS-CoV-2 infectivity or may lead to the possibility to multiply in the host. Therefore, it is advisable that future studies that experimentally inoculate this virus in cattle carefully select the animals in the experimental design based on SAP alleles in this gene.

Other host genes might be also involved in the infection mechanisms of CoV in cattle as well as in other mammalian species, as suggested by gene expression analyses in humans [63]. The genetic characterization within the cattle species of the selected host genes and of other genes that may emerge as relevant in CoV infections in future studies have the potential to identify putative functional markers that should be added in SNP arrays for facilitate implementation of genomic selection programs in cattle. In this way, it could facilitate the possibility to implement genomic selection plans, thereby aiming to increase genetic resistance to virus infections in cattle populations.

## 5. Conclusions

We presented a survey of genetic variants in coronavirus-related genes across *Bos taurus* populations. As a medium-term perspective, the present results can be used to better evaluate the basic biological mechanisms of CoV infection in cattle, starting from individual differences determined by variants in the host genome. As a long-term perspective, the results could be useful in defining breeding programs aimed at obtaining genetic improvement for disease resistance in cattle and new conservation strategies of cattle genetic resources. These elements can be included in a comprehensive “One Health” approach against CoV, whereby the genetic diversity of the host is not neglected in the risk assessment analysis. Comparative data across species could also be useful in better understanding the basic biological mechanisms of coronavirus–host interactions and disease progression. Genetic variability in farm animals could complement relevant information available about humans and other species in this context.

## Figures and Tables

**Table 1:** Number of variants and single amino acid polymorphisms (SAP) identified in the six coronavirus-related genes studied in the *Bos* genus. Ensembl and UniprotKB accession numbers of these genes are reported.

Gene	Ensembl Transcript	UniprotKB ACC	N. of Variants from WGS ^1^	N. of SAP from WGS ^2^	N. of SAP from Ensembl
*ACE2*	ENSBTAT00000048730.4	A0A452DJE0	1251 (358)	15 (4 + 6 + 5)	155
*ANPEP*	ENSBTAT00000068383.1	A0A3Q1MB09	464 (112)	45 (19 + 18 + 8)	73
*CEACAM1*	ENSBTAT00000069303.1	A0A3Q1MQ27	723 (212)	62 (41 + 7 + 14)	104
*DPP4*	ENSBTAT00000056886.3.1	P81425	2270 (716)	18 (3 + 9 + 6)	67
*FURIN*	ENSBTAT00000072776.1	B0JYR0	249 (71)	30 (9 + 13 + 8)	219
*TMPRSS2*	ENSBTAT00000012036.5.2	A2VDV7	1608 (526)	10 (6 + 1 + 3)	90
		Total	6565 (1995)	180 (82 + 54 + 44)	708

^1^ Total number of identified variants from whole genome sequencing (WGS). Round brackets: number of variants present in at least one *B. taurus* population; ^2^ Total number of variants affecting the protein sequence. Round brackets: SAP identified in at least two *Bos taurus* breeds + SAP private for *B. taurus* breeds + SAP identified only in *B. t. indicus*, *B. javanicus* and *B. grunniens* animals.

## Data Availability

Sequencing datasets are available in the EMBL-EBI European Nucleotide Archive (ENA) repository (http://www.ebi.ac.uk/ena; accessed on 14 September 2020). Sequencing projects and sample numbers are fully provided in Table S1.

## Supplementary Materials

The following supporting information can be downloaded at: https://www.mdpi.com/article/10.3390/ani12070838/s1, Figure S1: Principal Component Analysis of 34 *Bos* populations based on the allele frequencies of variants detected in the six coronavirus-related genes and present in at least one *B. taurus* population (n. 1995 variants). Table S1: Dataset used in the present study. Table S2: Genes involved in coronavirus infection investigated in the present study. Table S3: Variants (n. 82) identified in at least one B. taurus population. Table S4: ACE2 residues critical for protein function and coronavirus pathogenesis [30,31,56,57,64,65,66,67,68,69,70,71]. Table S5: ANPEP residues critical for protein function and coronavirus pathogenesis [47,72,73,74,75,76]. Table S6: CEACAM1 residues critical for protein function and coronavirus pathogenesis [25,77,78]. Table S7: DPP4 residues critical for protein function and coronavirus pathogenesis [71,79,80,81,82,83,84,85]. Table S8: FURIN residues critical for protein function and coronavirus pathogenesis. Table S9: TMPRSS2 residues critical for protein function and coronavirus pathogenesis [30,32,86].
