# Supplementary material for: One Health and Cattle Genetic Resources: Mining More than 500 Cattle Genomes to Identify Variants in Candidate Genes Potentially Affecting Coronavirus Infections
Source: Animals (Basel). 2022 Mar 26;12(7):838. doi: 10.3390/ani12070838 (PMC8997118; doi:10.3390/ani12070838)
Supplement: Supplementary file 1 [file animals-12-00838-s001.zip › animals-1567687-supplementary.pdf]

animals

MDPI

Supplementary Material

One Health and Cattle Genetic Resources: Mining More Than 500 Cattle Genomes to Identify Variants in Candidate Genes Potentially Affecting Coronavirus Infections

Samuele Bovo [1], Giuseppina Schiavo [1], Luca Fontanesi [1,*]

[1] Department of Agricultural and Food Sciences, University of Bologna, Viale Giuseppe Fanin 46, 40127 Bo-logna, Italy; samuele.bovo@unibo.it (S.B.); giuseppina.schiavo2@unibo.it (G.S.)

* Correspondence: luca.fontanesi@unibo.it (L.F); Tel.: +39 051 2096535

Figure S1. Principal Component Analysis of 34 *Bos* populations based on the allele frequencies of variants detected in the six coronavirus-related genes and present in at least one *B. taurus* population (n. 1,995 variants). The first two principal components (PC) are reported.

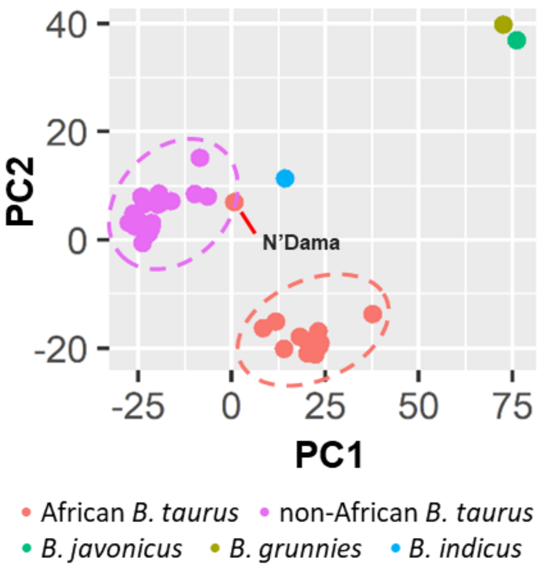

animals

MDPI

Table S1. Dataset used in the present study.

Scientific name	Taxid	ENA Sample accession	ENA Project accession	Population	Origin	No. of sequenced reads	Genome coverage (%)	Sequencing Depth (x)
Bos taurus	9913	SAMEA19309918	PRJEB18113	Simmental	non-African	407615764	99.6	22.8
Bos taurus	9913	SAMEA19317418	PRJEB18113	Holstein	non-African	341299966	99.6	18.8
Bos taurus	9913	SAMEA19318168	PRJEB18113	Holstein	non-African	420446422	99.6	26.3
Bos taurus	9913	SAMEA19325668	PRJEB18113	Holstein	non-African	428854832	99.6	24.0
Bos taurus	9913	SAMEA19846918	PRJEB18113	Holstein	non-African	394861020	99.5	14.9
Bos taurus	9913	SAMEA19847668	PRJEB18113	Brown Swiss (Bruna svizzera)	non-African	507873114	99.6	19.1
Bos taurus	9913	SAMEA19852168	PRJEB18113	Simmental	non-African	476511124	99.6	18.3
Bos taurus	9913	SAMEA19852918	PRJEB18113	Simmental	non-African	975930222	99.6	37.2
Bos taurus	9913	SAMEA19853668	PRJEB18113	Simmental	non-African	529681342	99.5	20.2
Bos taurus	9913	SAMEA19874668	PRJEB18113	Holstein	non-African	335302304	99.6	18.8
Bos taurus	9913	SAMEA19876918	PRJEB18113	Holstein	non-African	308017776	98.8	11.8
Bos taurus	9913	SAMEA32983168	PRJEB18113	Holstein	non-African	355059610	99.6	19.9
Bos taurus	9913	SAMEA32983918	PRJEB18113	Holstein	non-African	353736292	99.5	13.4
Bos taurus	9913	SAMEA32984668	PRJEB18113	Holstein	non-African	401208200	99.6	19.8
Bos taurus	9913	SAMEA32988418	PRJEB18113	Romagnola	non-African	632442748	99.6	27.3
Bos taurus	9913	SAMEA32989168	PRJEB18113	Romagnola	non-African	437153118	99.6	20.6
Bos taurus	9913	SAMEA32990668	PRJEB18113	Holstein	non-African	430988552	99.6	20.1
Bos taurus	9913	SAMEA32991418	PRJEB18113	Holstein	non-African	271523334	99.2	10.4
Bos taurus	9913	SAMEA32992168	PRJEB18113	Holstein	non-African	527795878	99.6	33.0
Bos taurus	9913	SAMEA32995168	PRJEB18113	Holstein	non-African	553519566	99.6	23.0
Bos taurus	9913	SAMEA32995918	PRJEB18113	Holstein	non-African	533324470	99.6	29.4

Bos taurus	9913	SAMEA32996668	PRJEB18113	Holstein	non-African	541558950	99.6	28.4
Bos taurus	9913	SAMEA32998168	PRJEB18113	Chianina	non-African	566030224	99.6	31.2
Bos taurus	9913	SAMEA32998918	PRJEB18113	Holstein	non-African	490158580	99.6	27.2
Bos taurus	9913	SAMEA32999668	PRJEB18113	Chianina	non-African	426104820	99.6	23.7
Bos taurus	9913	SAMEA33000418	PRJEB18113	Holstein	non-African	619737034	99.6	36.9
Bos taurus	9913	SAMEA33004168	PRJEB18113	Holstein	non-African	337796554	99.6	21.1
Bos taurus	9913	SAMEA33004918	PRJEB18113	Simmental	non-African	423140032	99.6	23.6
Bos taurus	9913	SAMEA33668668	PRJEB18113	Chianina	non-African	386160442	99.6	21.4
Bos taurus	9913	SAMEA4644726	PRJEB18113	Holstein	non-African	321756358	99.1	12.5
Bos taurus	9913	SAMEA4644729	PRJEB18113	Holstein	non-African	334831432	99.2	13.0
Bos taurus	9913	SAMEA4644732	PRJEB18113	Holstein	non-African	368909172	99.4	14.3
Bos taurus	9913	SAMEA4644736	PRJEB18113	Holstein	non-African	334188110	98.5	12.8
Bos taurus	9913	SAMEA4644737	PRJEB18113	Holstein	non-African	315100578	99.6	19.7
Bos taurus	9913	SAMEA4644738	PRJEB18113	Holstein	non-African	326126082	99.6	18.2
Bos taurus	9913	SAMEA4644746	PRJEB18113	Holstein	non-African	329128266	99.3	12.9
Bos taurus	9913	SAMEA4644748	PRJEB18113	Holstein	non-African	434268400	99.6	26.9
Bos taurus	9913	SAMEA4644753	PRJEB18113	Holstein	non-African	448095426	99.6	27.8
Bos taurus	9913	SAMEA4644759	PRJEB18113	Holstein	non-African	292721478	99.6	16.3
Bos taurus	9913	SAMEA4644761	PRJEB18113	Holstein	non-African	269328566	99.5	14.9
Bos taurus	9913	SAMEA4644766	PRJEB18113	Brown Swiss (Bruna svizzera)	non-African	408734786	99.6	22.8
Bos taurus	9913	SAMEA4644769	PRJEB18113	Brown Swiss (Bruna svizzera)	non-African	294530942	73.1	16.2
Bos taurus	9913	SAMEA5159761	PRJEB18113	Brown Swiss (Bruna svizzera)	non-African	474078400	99.3	18.3
Bos taurus	9913	SAMEA5159778	PRJEB18113	Brown Swiss (Bruna svizzera)	non-African	380015822	99.3	14.9
Bos taurus	9913	SAMEA5159802	PRJEB18113	Holstein	non-African	327781418	99.3	12.7

Bos taurus	9913	SAMEA5159804	PRJEB18113	Holstein	non-African	349678832	99.2	13.6
Bos taurus	9913	SAMEA5159808	PRJEB18113	Holstein	non-African	330016346	99.5	20.7
Bos taurus	9913	SAMEA5159809	PRJEB18113	Holstein	non-African	429432888	99.6	25.1
Bos taurus	9913	SAMEA5159810	PRJEB18113	Holstein	non-African	309139506	99.6	19.3
Bos taurus	9913	SAMEA5159813	PRJEB18113	Holstein	non-African	430988552	99.6	20.1
Bos taurus	9913	SAMEA5159814	PRJEB18113	Holstein	non-African	533324470	99.6	29.4
Bos taurus	9913	SAMEA5159815	PRJEB18113	Holstein	non-African	360963054	99.4	13.9
Bos taurus	9913	SAMEA5159816	PRJEB18113	Holstein	non-African	489924500	99.6	30.2
Bos taurus	9913	SAMEA5159818	PRJEB18113	Holstein	non-African	343124662	99.6	21.2
Bos taurus	9913	SAMEA5159819	PRJEB18113	Holstein	non-African	321761154	99.1	12.5
Bos taurus	9913	SAMEA5159820	PRJEB18113	Holstein	non-African	516344046	99.6	31.9
Bos taurus	9913	SAMEA5159821	PRJEB18113	Holstein	non-African	356137794	99.6	19.9
Bos taurus	9913	SAMEA5159822	PRJEB18113	Holstein	non-African	412287140	99.6	25.7
Bos taurus	9913	SAMEA5159828	PRJEB18113	Holstein	non-African	338477598	99.6	18.7
Bos taurus	9913	SAMEA5159833	PRJEB18113	Holstein	non-African	508114464	99.6	30.6
Bos taurus	9913	SAMEA5159834	PRJEB18113	Holstein	non-African	391347710	99.6	23.3
Bos taurus	9913	SAMEA5159836	PRJEB18113	Piedmontese	non-African	506218766	99.6	31.1
Bos taurus	9913	SAMEA5159847	PRJEB18113	Brown Swiss (Bruna svizzera)	non-African	474238470	99.6	29.5
Bos taurus	9913	SAMEA5159849	PRJEB18113	Original Braunvieh (Bruna)	non-African	493514772	99.6	30.0
Bos taurus	9913	SAMEA5159850	PRJEB18113	Original Braunvieh (Bruna)	non-African	519836264	99.6	31.5
Bos taurus	9913	SAMEA5159853	PRJEB18113	Brown Swiss (Bruna svizzera)	non-African	576587084	99.6	35.7
Bos taurus	9913	SAMEA5159857	PRJEB18113	Holstein	non-African	522162460	99.6	31.2
Bos taurus	9913	SAMEA5159858	PRJEB18113	Holstein	non-African	384527000	99.6	23.2
Bos taurus	9913	SAMEA5159859	PRJEB18113	Holstein	non-African	473053576	99.6	28.3
Bos taurus	9913	SAMEA5159861	PRJEB18113	Brown Swiss (Bruna svizzera)	non-African	464047572	99.6	27.3

Bos taurus	9913	SAMEA5159881	PRJEB18113	Holstein	non-African	316820754	99	12.2
Bos taurus	9913	SAMEA5159884	PRJEB18113	Holstein	non-African	572904122	99.6	35.0
Bos taurus	9913	SAMEA5159886	PRJEB18113	Original Braunvieh (Bruna)	non-African	672627258	99.6	41.2
Bos taurus	9913	SAMEA5159887	PRJEB18113	Tyrolean Grey (Grigio alpina)	non-African	466472678	99.6	27.9
Bos taurus	9913	SAMEA5159888	PRJEB18113	Tyrolean Grey (Grigio alpina)	non-African	459426910	99.6	28.4
Bos taurus	9913	SAMEA5159889	PRJEB18113	Tyrolean Grey (Grigio alpina)	non-African	443290092	99.6	27.4
Bos taurus	9913	SAMEA5160021	PRJEB18113	Holstein	non-African	281524054	98.5	10.8
Bos taurus	9913	SAMEA5160153	PRJEB18113	Simmental	non-African	634505698	99.6	37.5
Bos taurus	9913	SAMEA5415484	PRJEB18113	Holstein	non-African	324907910	99.6	20.2
Bos taurus	9913	SAMEA5415492	PRJEB18113	Holstein	non-African	396390048	99.6	22.0
Bos taurus	9913	SAMEA5415494	PRJEB18113	Holstein	non-African	455481366	99.6	25.3
Bos taurus	9913	SAMEA5415496	PRJEB18113	Holstein	non-African	213934654	99.5	11.8
Bos taurus	9913	SAMEA5415500	PRJEB18113	Holstein	non-African	388743922	99.6	21.6
Bos taurus	9913	SAMEA5415501	PRJEB18113	Holstein	non-African	319224558	99.2	12.4
Bos taurus	9913	SAMEA5415503	PRJEB18113	Holstein	non-African	579721386	99.6	34.7
Bos taurus	9913	SAMEA5564719	PRJEB18113	Simmental	non-African	461980954	99.6	29.0
Bos taurus	9913	SAMEA5564721	PRJEB18113	Simmental	non-African	403960668	99.6	25.0
Bos taurus	9913	SAMEA5564723	PRJEB18113	Simmental	non-African	496628660	99.6	30.7
Bos taurus	9913	SAMEA5564730	PRJEB18113	Tyrolean Grey (Grigio alpina)	non-African	473823044	99.6	27.8
Bos taurus	9913	SAMEA5564732	PRJEB18113	Tyrolean Grey (Grigio alpina)	non-African	436564384	99.6	26.1
Bos taurus	9913	SAMEA5564734	PRJEB18113	Tyrolean Grey (Grigio alpina)	non-African	452924566	99.6	27.7

Bos taurus	9913	SAMEA5564737	PRJEB18113	Tyrolean Grey (Grigio alpina)	non-African	423791974	99.6	25.2
Bos taurus	9913	SAMEA5714972	PRJEB18113	Original Braunvieh (Bruna)	non-African	588837408	99.6	36.4
Bos taurus	9913	SAMEA5714974	PRJEB18113	Original Braunvieh (Bruna)	non-African	484822916	99.6	28.9
Bos taurus	9913	SAMEA5714975	PRJEB18113	Original Braunvieh (Bruna)	non-African	415712800	99.6	25.9
Bos taurus	9913	SAMEA5714978	PRJEB18113	Holstein	non-African	256531092	99.5	14.1
Bos taurus	9913	SAMEA6528881	PRJEB18113	Romagnola	non-African	385891112	99.6	23.8
Bos taurus	9913	SAMEA6528882	PRJEB18113	Romagnola	non-African	382869688	99.6	23.7
Bos taurus	9913	SAMEA6528883	PRJEB18113	Romagnola	non-African	416140380	99.6	25.5
Bos taurus	9913	SAMEA6528884	PRJEB18113	Romagnola	non-African	447472396	99.6	27.5
Bos taurus	9913	SAMEA6528885	PRJEB18113	Romagnola	non-African	411272862	99.6	25.1
Bos taurus	9913	SAMEA6528886	PRJEB18113	Original Braunvieh (Bruna)	non-African	434174082	99.6	27.4
Bos taurus	9913	SAMEA6528887	PRJEB18113	Original Braunvieh (Bruna)	non-African	401239868	99.2	23.4
Bos taurus	9913	SAMEA6528891	PRJEB18113	Original Braunvieh (Bruna)	non-African	434883262	99.6	27.1
Bos taurus	9913	SAMEA6528892	PRJEB18113	Original Braunvieh (Bruna)	non-African	383256970	99.6	23.9
Bos taurus	9913	SAMEA6528896	PRJEB18113	Holstein	non-African	401208200	99.6	19.8
Bos taurus	9913	SAMEA6528899	PRJEB18113	Holstein	non-African	255262022	99.3	9.7
Bos taurus	9913	SAMEA6528902	PRJEB18113	Holstein	non-African	546661962	99.6	32.1
Bos taurus	9913	SAMEA6528903	PRJEB18113	Holstein	non-African	352365600	99.2	13.6
Bos taurus	9913	SAMEA6528904	PRJEB18113	Holstein	non-African	429771510	99.6	25.9
Bos taurus	9913	SAMEA6528905	PRJEB18113	Holstein	non-African	511799706	99.6	30.6
Bos taurus	9913	SAMEA6528907	PRJEB18113	Holstein	non-African	467126056	99.6	28.4
Bos taurus	9913	SAMEA6528908	PRJEB18113	Holstein	non-African	334504408	99.6	18.5
Bos taurus	9913	SAMEA6528909	PRJEB18113	Holstein	non-African	646249702	99.6	39.7
Bos taurus	9913	SAMEA7015110	PRJEB18113	Holstein	non-African	310812448	99.3	12.2
Bos taurus	9913	SAMEA7015112	PRJEB18113	Holstein	non-African	226214558	99.5	13.8
Bos taurus	9913	SAMEA7015113	PRJEB18113	Holstein	non-African	424409424	99.6	25.5
Bos taurus	9913	SAMEA7015115	PRJEB18113	Holstein	non-African	235300828	99.6	13.2

Bos taurus	9913	SAMEA7015119	PRJEB18113	Tyrolean Grey (Grigio alpina)	non-African	465314596	99.6	28.7
Bos indicus	9915	SAMEA7195632	PRJEB39924	Djakkore cattle	Bos indicus	739246388	99.6	33.9
Bos taurus	9913	SAMN01915332	PRJNA176557	angus	non-African	258856624	99.1	8.6
Bos taurus	9913	SAMN01915333	PRJNA176557	Red Angus	non-African	278819588	99.4	10.8
Bos taurus	9913	SAMN01915334	PRJNA176557	Red Angus	non-African	290114850	99.4	11.1
Bos taurus	9913	SAMN01915335	PRJNA176557	angus	non-African	243284180	99.1	8.2
Bos taurus	9913	SAMN01915336	PRJNA176557	angus	non-African	290820242	99.4	11.0
Bos taurus	9913	SAMN01915337	PRJNA176557	angus	non-African	277427602	99.4	10.6
Bos taurus	9913	SAMN01915338	PRJNA176557	Red Angus	non-African	247271114	99.2	9.4
Bos taurus	9913	SAMN01915339	PRJNA176557	angus	non-African	282005764	99.4	10.6
Bos taurus	9913	SAMN01915340	PRJNA176557	Red Angus	non-African	292717726	99.4	11.1
Bos taurus	9913	SAMN01915341	PRJNA176557	angus	non-African	270493868	99.3	10.1
Bos taurus	9913	SAMN01915342	PRJNA176557	Gelbvieh	non-African	325002814	99.5	12.2
Bos taurus	9913	SAMN01915343	PRJNA176557	Simmental	non-African	293334302	99.4	10.9
Bos taurus	9913	SAMN01915344	PRJNA176557	Gelbvieh	non-African	275491206	99.4	10.4
Bos taurus	9913	SAMN01915345	PRJNA176557	Piedmontese	non-African	256624306	99.3	10.1
Bos taurus	9913	SAMN01915347	PRJNA176557	Red Angus	non-African	300481176	99.4	11.3
Bos taurus	9913	SAMN01915348	PRJNA176557	angus	non-African	316202084	99.4	10.6
Bos taurus	9913	SAMN01915349	PRJNA176557	Piedmontese	non-African	298486344	99.4	11.0
Bos taurus	9913	SAMN01915350	PRJNA176557	Red Angus	non-African	316702266	99.3	11.0
Bos taurus	9913	SAMN01915351	PRJNA176557	Simmental	non-African	301215438	99.3	10.8
Bos taurus	9913	SAMN01915352	PRJNA176557	Charolais	non-African	301625296	99.4	11.3
Bos taurus	9913	SAMN01915353	PRJNA176557	Hereford	non-African	323451972	99.5	12.1
Bos taurus	9913	SAMN01915354	PRJNA176557	Charolais	non-African	296188976	99.4	11.2
Bos taurus	9913	SAMN01915355	PRJNA176557	Blonde d'Aquitaine	non-African	253614070	99.3	9.9
Bos taurus	9913	SAMN01915356	PRJNA176557	Red Angus	non-African	291827884	99.4	11.3
Bos taurus	9913	SAMN01915357	PRJNA176557	Limosine	non-African	282133948	99.4	11.0

Bos taurus	9913	SAMN01915359	PRJNA176557	Red Angus	non-African	287916438	99.4	11.2
Bos taurus	9913	SAMN01915360	PRJNA176557	Limosine	non-African	285644040	99.5	11.3
Bos taurus	9913	SAMN01920653	PRJNA176557	Limosine	non-African	274812974	99.4	10.6
Bos taurus	9913	SAMN02142982	PRJNA176557	angus	non-African	280000364	98.6	10.2
Bos taurus	9913	SAMN02142983	PRJNA176557	angus	non-African	258446298	99.3	9.5
Bos taurus	9913	SAMN02142984	PRJNA176557	angus	non-African	271104266	99.2	10.3
Bos taurus	9913	SAMN02142985	PRJNA176557	angus	non-African	288000226	99.4	11.2
Bos taurus	9913	SAMN02142986	PRJNA176557	angus	non-African	284905390	99.4	11.0
Bos taurus	9913	SAMN02142987	PRJNA176557	angus	non-African	263913032	99.2	9.9
Bos taurus	9913	SAMN02142988	PRJNA176557	angus	non-African	277161238	99.3	10.7
Bos taurus	9913	SAMN02142989	PRJNA176557	angus	non-African	274968962	99.3	10.3
Bos taurus	9913	SAMN02142990	PRJNA176557	angus	non-African	288419588	99.4	10.9
Bos taurus	9913	SAMN02142991	PRJNA176557	Charolais	non-African	265763148	99	10.4
Bos taurus	9913	SAMN02142992	PRJNA176557	Charolais	non-African	297044452	99.2	11.6
Bos taurus	9913	SAMN02142993	PRJNA176557	Charolais	non-African	295817318	99.1	11.7
Bos taurus	9913	SAMN02142994	PRJNA176557	Charolais	non-African	283296916	99	10.1
Bos taurus	9913	SAMN02142995	PRJNA176557	Charolais	non-African	283462478	99.3	10.5
Bos taurus	9913	SAMN02142996	PRJNA176557	Hereford	non-African	265491034	99.3	10.5
Bos taurus	9913	SAMN02142997	PRJNA176557	Hereford	non-African	314455760	99.5	12.0
Bos taurus	9913	SAMN02142998	PRJNA176557	angus	non-African	374708712	99.4	13.4
Bos taurus	9913	SAMN02142999	PRJNA176557	Red Angus	non-African	209861616	98.5	8.2
Bos taurus	9913	SAMN02143000	PRJNA176557	angus	non-African	200994378	98.9	7.7
Bos taurus	9913	SAMN02143001	PRJNA176557	Red Angus	non-African	172018886	98.3	6.7
Bos taurus	9913	SAMN02143002	PRJNA176557	Hereford	non-African	216901344	99.1	8.3
Bos taurus	9913	SAMN02143003	PRJNA176557	Hereford	non-African	178495950	98.7	6.6
Bos taurus	9913	SAMN02143004	PRJNA176557	Hereford	non-African	158311854	97.9	5.9
Bos taurus	9913	SAMN02671560	PRJNA238491	Holstein	non-African	215624044	52.9	8.1
Bos taurus	9913	SAMN02671564	PRJNA238491	Holstein	non-African	592544266	99.5	21.0

Bos taurus	9913	SAMN02671567	PRJNA238491	Holstein	non-African	705993154	99.5	25.9
Bos taurus	9913	SAMN02671582	PRJNA238491	Holstein	non-African	167807814	94.9	5.0
Bos taurus	9913	SAMN02671586	PRJNA238491	Holstein	non-African	149359872	96	5.8
Bos taurus	9913	SAMN02671587	PRJNA238491	Holstein	non-African	537792432	99.1	13.4
Bos taurus	9913	SAMN02671588	PRJNA238491	Holstein	non-African	462928796	99.3	17.3
Bos taurus	9913	SAMN02671592	PRJNA238491	Holstein	non-African	850584834	99.6	29.1
Bos taurus	9913	SAMN02671593	PRJNA238491	Holstein	non-African	560621108	99.4	17.5
Bos taurus	9913	SAMN02671594	PRJNA238491	Holstein	non-African	517540398	99.5	17.8
Bos taurus	9913	SAMN02671596	PRJNA238491	Holstein	non-African	320696208	99.1	11.0
Bos taurus	9913	SAMN02671597	PRJNA238491	Jersey	non-African	235303922	98.7	9.0
Bos taurus	9913	SAMN02671598	PRJNA238491	Jersey	non-African	223894648	98.7	8.6
Bos taurus	9913	SAMN02671599	PRJNA238491	Jersey	non-African	251477070	98.9	9.7
Bos taurus	9913	SAMN02671600	PRJNA238491	Jersey	non-African	187391086	96.5	7.4
Bos taurus	9913	SAMN02671601	PRJNA238491	Jersey	non-African	366807426	99.4	14.1
Bos taurus	9913	SAMN02671602	PRJNA238491	Jersey	non-African	173404692	96	6.8
Bos taurus	9913	SAMN02671603	PRJNA238491	Jersey	non-African	193235446	97.9	7.5
Bos taurus	9913	SAMN02671606	PRJNA238491	Jersey	non-African	346974152	98.6	11.4
Bos taurus	9913	SAMN02671607	PRJNA238491	Jersey	non-African	293932954	98.9	10.6
Bos taurus	9913	SAMN02671608	PRJNA238491	Jersey	non-African	191809594	97.9	7.4
Bos taurus	9913	SAMN02671609	PRJNA238491	Jersey	non-African	312076916	98.1	11.9
Bos taurus	9913	SAMN02671610	PRJNA238491	Jersey	non-African	289109764	99.1	10.4
Bos taurus	9913	SAMN02671611	PRJNA238491	Jersey	non-African	271353624	99.3	10.9
Bos taurus	9913	SAMN02671625	PRJNA238491	Simmental	non-African	163300816	97.2	6.2
Bos taurus	9913	SAMN02671627	PRJNA238491	Simmental	non-African	226108574	98.5	8.5
Bos taurus	9913	SAMN02671628	PRJNA238491	Simmental	non-African	161645566	97.5	6.1
Bos taurus	9913	SAMN02671629	PRJNA238491	Simmental	non-African	284066080	99.1	10.9
Bos taurus	9913	SAMN02671631	PRJNA238491	Simmental	non-African	156504100	97.4	6.0
Bos taurus	9913	SAMN02671634	PRJNA238491	Simmental	non-African	260887632	99.1	9.6

Bos taurus	9913	SAMN02671635	PRJNA238491	Simmental	non-African	217813826	98.9	8.3
Bos taurus	9913	SAMN02671637	PRJNA238491	Simmental	non-African	131222468	93.4	5.1
Bos taurus	9913	SAMN02671640	PRJNA238491	Simmental	non-African	295019312	99.2	11.2
Bos taurus	9913	SAMN02671642	PRJNA238491	Simmental	non-African	181317924	97.9	6.9
Bos taurus	9913	SAMN02671643	PRJNA238491	Simmental	non-African	175962316	97.4	6.6
Bos taurus	9913	SAMN02671644	PRJNA238491	Simmental	non-African	160031528	94.7	6.2
Bos taurus	9913	SAMN02671645	PRJNA238491	Simmental	non-African	160664402	97.6	6.1
Bos taurus	9913	SAMN02671646	PRJNA238491	Simmental	non-African	206798248	97.7	7.7
Bos taurus	9913	SAMN02671647	PRJNA238491	Simmental	non-African	173517682	93.9	6.8
Bos taurus	9913	SAMN02671648	PRJNA238491	Simmental	non-African	208220458	97.7	7.7
Bos taurus	9913	SAMN02671649	PRJNA238491	Simmental	non-African	189777736	97.8	7.1
Bos taurus	9913	SAMN02671650	PRJNA238491	Simmental	non-African	172879558	97.7	6.6
Bos taurus	9913	SAMN02671651	PRJNA238491	Simmental	non-African	150609618	97	6.0
Bos taurus	9913	SAMN02671652	PRJNA238491	Simmental	non-African	251149450	99	9.4
Bos taurus	9913	SAMN02671653	PRJNA238491	Simmental	non-African	256765728	99.1	9.8
Bos taurus	9913	SAMN02671654	PRJNA238491	Simmental	non-African	275027722	99.2	10.3
Bos taurus	9913	SAMN02671656	PRJNA238491	Simmental	non-African	160546618	96.9	6.1
Bos taurus	9913	SAMN02671657	PRJNA238491	Simmental	non-African	208168550	97	7.7
Bos taurus	9913	SAMN02671658	PRJNA238491	Simmental	non-African	160463852	97.7	6.1
Bos taurus	9913	SAMN02671659	PRJNA238491	Simmental	non-African	160443150	96.8	5.8
Bos taurus	9913	SAMN02671663	PRJNA238491	Simmental	non-African	178154978	97.9	6.8
Bos taurus	9913	SAMN02671664	PRJNA238491	Simmental	non-African	342410932	99.2	11.7
Bos taurus	9913	SAMN02671665	PRJNA238491	Simmental	non-African	225043190	98.6	8.5
Bos taurus	9913	SAMN02671666	PRJNA238491	Simmental	non-African	207640612	97.8	7.8
Bos taurus	9913	SAMN02671667	PRJNA238491	Simmental	non-African	287051728	99.1	11.0
Bos taurus	9913	SAMN02839601	PRJNA176557	Gelbvieh	non-African	364576332	99.6	14.0
Bos taurus	9913	SAMN02839602	PRJNA176557	Gelbvieh	non-African	444000000	99.6	15.9
Bos taurus	9913	SAMN02839603	PRJNA176557	Gelbvieh	non-African	140938730	97.6	5.4

Bos taurus	9913	SAMN02839605	PRJNA176557	Hereford	non-African	467195796	99.6	15.6
Bos taurus	9913	SAMN02839606	PRJNA176557	Hereford	non-African	110744840	96.2	4.2
Bos taurus	9913	SAMN02839608	PRJNA176557	Charolais	non-African	359289266	99.6	13.7
Bos taurus	9913	SAMN02839609	PRJNA176557	Charolais	non-African	358565250	99.5	13.6
Bos taurus	9913	SAMN02839610	PRJNA176557	Charolais	non-African	478016152	99.6	18.0
Bos taurus	9913	SAMN02839611	PRJNA176557	Charolais	non-African	429689022	99.6	15.8
Bos taurus	9913	SAMN02839612	PRJNA176557	Charolais	non-African	371812724	99.6	14.2
Bos taurus	9913	SAMN02841107	PRJNA176557	Gelbvieh	non-African	381157242	99.5	14.7
Bos taurus	9913	SAMN02841110	PRJNA176557	Hereford	non-African	409506512	99.5	14.9
Bos taurus	9913	SAMN02841111	PRJNA176557	Hereford	non-African	513396908	99.6	19.8
Bos taurus	9913	SAMN02841112	PRJNA176557	Hereford	non-African	544570838	99.6	20.4
Bos taurus	9913	SAMN02841115	PRJNA176557	Hereford	non-African	535374584	99.6	20.4
Bos taurus	9913	SAMN02841117	PRJNA176557	Holstein	non-African	369283064	99.5	14.7
Bos taurus	9913	SAMN02841118	PRJNA176557	Holstein	non-African	378403475	99.6	14.6
Bos taurus	9913	SAMN02842700	PRJNA176557	Charolais	non-African	372739694	99.6	14.6
Bos taurus	9913	SAMN02842701	PRJNA176557	Holstein	non-African	464414926	99.6	17.8
Bos taurus	9913	SAMN02842704	PRJNA176557	Charolais	non-African	315131546	99.5	12.0
Bos taurus	9913	SAMN02842705	PRJNA176557	Charolais	non-African	345631606	99.6	13.4
Bos taurus	9913	SAMN02842706	PRJNA176557	Charolais	non-African	405230006	99.6	15.4
Bos taurus	9913	SAMN02842707	PRJNA176557	Gelbvieh	non-African	355868842	99.6	13.8
Bos taurus	9913	SAMN02842708	PRJNA176557	Gelbvieh	non-African	422365644	99.6	15.4
Bos taurus	9913	SAMN02842711	PRJNA176557	Holstein	non-African	469000090	99.6	17.9
Bos taurus	9913	SAMN02843056	PRJNA176557	Charolais	non-African	427430922	99.5	17.0
Bos taurus	9913	SAMN02843058	PRJNA176557	Charolais	non-African	321002120	99.5	12.5
Bos taurus	9913	SAMN02843059	PRJNA176557	Gelbvieh	non-African	339250526	99.5	13.2
Bos taurus	9913	SAMN02843060	PRJNA176557	Gelbvieh	non-African	228505608	99.2	8.7
Bos taurus	9913	SAMN02843062	PRJNA176557	Gelbvieh	non-African	372115096	99.6	14.3
Bos taurus	9913	SAMN02843064	PRJNA176557	Maine Anjou	non-African	327934402	99.5	12.5

Bos taurus	9913	SAMN02843065	PRJNA176557	Maine Anjou	non-African	412442214	99.3	14.9
Bos taurus	9913	SAMN02843066	PRJNA176557	Charolais	non-African	429570610	99.5	16.6
Bos taurus	9913	SAMN02843067	PRJNA176557	Gelbvieh	non-African	89817940	92.7	3.5
Bos taurus	9913	SAMN02843069	PRJNA176557	Maine Anjou	non-African	346541988	99.5	13.3
Bos taurus	9913	SAMN02843070	PRJNA176557	Gelbvieh	non-African	381956714	95.8	10.1
Bos taurus	9913	SAMN02843071	PRJNA176557	Gelbvieh	non-African	433378046	99.6	16.3
Bos taurus	9913	SAMN02843072	PRJNA176557	Gelbvieh	non-African	539772282	99.6	20.4
Bos taurus	9913	SAMN02843073	PRJNA176557	Gelbvieh	non-African	442526202	99.6	16.7
Bos taurus	9913	SAMN02843074	PRJNA176557	Gelbvieh	non-African	422990044	99.6	15.9
Bos taurus	9913	SAMN02843075	PRJNA176557	Gelbvieh	non-African	428308644	99.6	16.3
Bos taurus	9913	SAMN02843077	PRJNA176557	Gelbvieh	non-African	421748518	99.6	15.7
Bos taurus	9913	SAMN02843078	PRJNA176557	Gelbvieh	non-African	506709024	99.6	18.9
Bos taurus	9913	SAMN02843088	PRJNA176557	Gelbvieh	non-African	444163266	99.1	16.2
Bos taurus	9913	SAMN02843089	PRJNA176557	Gelbvieh	non-African	522945538	99.6	18.6
Bos taurus	9913	SAMN02843090	PRJNA176557	Hereford	non-African	377533034	99.5	14.2
Bos taurus	9913	SAMN02843092	PRJNA176557	Hereford	non-African	402043058	99.6	14.6
Bos taurus	9913	SAMN02843093	PRJNA176557	Hereford	non-African	343702008	99.5	12.2
Bos taurus	9913	SAMN02843094	PRJNA176557	Limosine	non-African	483313372	99.6	18.6
Bos taurus	9913	SAMN02843095	PRJNA176557	Limosine	non-African	485617914	99.6	18.6
Bos taurus	9913	SAMN02843096	PRJNA176557	Gelbvieh	non-African	430759700	99.6	16.1
Bos taurus	9913	SAMN02843097	PRJNA176557	Hereford	non-African	417311596	99.6	14.9
Bos taurus	9913	SAMN02843098	PRJNA176557	Hereford	non-African	454996380	99.6	16.6
Bos taurus	9913	SAMN02843099	PRJNA176557	Hereford	non-African	406083280	99.5	14.9
Bos taurus	9913	SAMN02843100	PRJNA176557	Maine Anjou	non-African	412547918	99.4	14.7
Bos taurus	9913	SAMN02843108	PRJNA176557	Charolais	non-African	384117084	99.6	14.8
Bos taurus	9913	SAMN02843109	PRJNA176557	Hereford	non-African	409976018	99.5	15.1
Bos taurus	9913	SAMN02843110	PRJNA176557	Holstein	non-African	526962010	99.6	20.4
Bos taurus	9913	SAMN02843112	PRJNA176557	Hereford	non-African	418270906	99.6	12.9

Bos taurus	9913	SAMN02843113	PRJNA176557	Hereford	non-African	471824856	99.6	14.8
Bos taurus	9913	SAMN02843120	PRJNA176557	Holstein	non-African	480938978	99.6	18.5
Bos taurus	9913	SAMN02843123	PRJNA176557	Charolais	non-African	349262216	99.5	13.6
Bos taurus	9913	SAMN02843124	PRJNA176557	Holstein	non-African	461749022	99.6	17.2
Bos taurus	9913	SAMN02843125	PRJNA176557	Holstein	non-African	465084086	99.6	17.4
Bos taurus	9913	SAMN02843127	PRJNA176557	Holstein	non-African	81196718	90.8	3.2
Bos taurus	9913	SAMN02843128	PRJNA176557	Holstein	non-African	89742154	92.6	3.5
Bos taurus	9913	SAMN02843129	PRJNA176557	Holstein	non-African	389386630	99.6	14.6
Bos taurus	9913	SAMN02843130	PRJNA176557	Holstein	non-African	400934886	99.6	15.3
Bos taurus	9913	SAMN02843132	PRJNA176557	Hereford	non-African	439291528	99.6	16.5
Bos taurus	9913	SAMN02843133	PRJNA176557	Hereford	non-African	412780220	99.6	15.3
Bos taurus	9913	SAMN02843134	PRJNA176557	Hereford	non-African	396799662	99.6	14.5
Bos taurus	9913	SAMN02843135	PRJNA176557	Hereford	non-African	398789472	99.6	14.7
Bos taurus	9913	SAMN02843136	PRJNA176557	Maine Anjou	non-African	377158690	99.4	13.7
Bos taurus	9913	SAMN02843137	PRJNA176557	Maine Anjou	non-African	347876774	99.5	12.9
Bos taurus	9913	SAMN02843138	PRJNA176557	Maine Anjou	non-African	343807302	99.5	13.2
Bos taurus	9913	SAMN02843139	PRJNA176557	Holstein	non-African	449911406	99.6	17.0
Bos taurus	9913	SAMN02843150	PRJNA176557	Gelbvieh	non-African	363849408	99.5	14.1
Bos taurus	9913	SAMN02843153	PRJNA176557	Gelbvieh	non-African	351014520	99.5	13.5
Bos taurus	9913	SAMN02843154	PRJNA176557	Holstein	non-African	464010006	99.6	17.9
Bos taurus	9913	SAMN02843155	PRJNA176557	Holstein	non-African	340628176	99.5	13.0
Bos taurus	9913	SAMN02843157	PRJNA176557	Holstein	non-African	432805756	99.6	16.0
Bos taurus	9913	SAMN02843158	PRJNA176557	Holstein	non-African	452503342	99.6	17.1
Bos taurus	9913	SAMN02843159	PRJNA176557	Holstein	non-African	446460452	99.6	16.9
Bos taurus	9913	SAMN02843161	PRJNA176557	Holstein	non-African	443586398	99.6	16.8
Bos taurus	9913	SAMN02843162	PRJNA176557	Holstein	non-African	446269774	99.6	16.8
Bos taurus	9913	SAMN02843163	PRJNA176557	Holstein	non-African	452795190	99.6	17.0
Bos taurus	9913	SAMN02843164	PRJNA176557	Holstein	non-African	352728780	99.5	13.4

Bos taurus	9913	SAMN02843165	PRJNA176557	Holstein	non-African	120098280	96.6	4.7
Bos taurus	9913	SAMN02843166	PRJNA176557	Holstein	non-African	512343314	99.6	19.8
Bos taurus	9913	SAMN02843167	PRJNA176557	Holstein	non-African	344574416	99.5	13.0
Bos taurus	9913	SAMN02843168	PRJNA176557	Holstein	non-African	408792892	99.6	16.0
Bos taurus	9913	SAMN02843169	PRJNA176557	Holstein	non-African	436866200	99.6	16.0
Bos taurus	9913	SAMN02843171	PRJNA176557	Holstein	non-African	448547520	99.6	17.3
Bos taurus	9913	SAMN02843172	PRJNA176557	Holstein	non-African	283972410	99.4	10.8
Bos taurus	9913	SAMN02843173	PRJNA176557	Holstein	non-African	248276194	99.4	9.7
Bos taurus	9913	SAMN02843174	PRJNA176557	Holstein	non-African	415587012	99.6	16.1
Bos taurus	9913	SAMN02843175	PRJNA176557	Holstein	non-African	288950082	99.4	11.1
Bos taurus	9913	SAMN02843176	PRJNA176557	Holstein	non-African	504150586	99.6	19.3
Bos taurus	9913	SAMN02843177	PRJNA176557	Holstein	non-African	492491024	99.6	19.1
Bos taurus	9913	SAMN02843178	PRJNA176557	Holstein	non-African	475434548	99.6	18.2
Bos taurus	9913	SAMN02843181	PRJNA176557	Holstein	non-African	342790830	99.5	12.9
Bos taurus	9913	SAMN02843182	PRJNA176557	Holstein	non-African	527367042	99.6	20.2
Bos taurus	9913	SAMN02941215	PRJNA256210	Piedmontese	non-African	360353880	99.6	13.6
Bos taurus	9913	SAMN02941216	PRJNA256210	Piedmontese	non-African	409883698	99.6	14.6
Bos taurus	9913	SAMN02941217	PRJNA256210	Piedmontese	non-African	421336880	99.6	14.7
Bos taurus	9913	SAMN02941218	PRJNA256210	Piedmontese	non-African	396765898	99.5	14.8
Bos taurus	9913	SAMN02941219	PRJNA256210	Piedmontese	non-African	417835594	99.6	15.1
Bos grunniens	30521	SAMN03766764	PRJNA285834	Bos grunniens	Bos grunniens	564969420	99.2	23.4
Bos grunniens	30521	SAMN03766790	PRJNA285834	Bos grunniens	Bos grunniens	615246732	99.1	22.1
Bos grunniens	30521	SAMN03766792	PRJNA285834	Bos grunniens	Bos grunniens	657837900	99	20.3
Bos taurus	9913	SAMN05788484	PRJNA343262	Angus	non-African	476113200	99.6	14.3
Bos taurus	9913	SAMN05788497	PRJNA343262	Angus	non-African	482404566	99.6	17.7

Bos taurus	9913	SAMN05788510	PRJNA343262	Angus	non-African	511526938	99.6	19.1
Bos taurus	9913	SAMN05788511	PRJNA343262	Angus	non-African	457108776	99.6	16.7
Bos taurus	9913	SAMN05788530	PRJNA343262	Angus	non-African	472468022	99.6	17.1
Bos taurus	9913	SAMN05788531	PRJNA343262	Hereford	non-African	461168748	99.6	17.0
Bos taurus	9913	SAMN05788534	PRJNA343262	Hereford	non-African	433586058	99.6	15.7
Bos taurus	9913	SAMN05788535	PRJNA343262	Hereford	non-African	453217078	99.6	16.4
Bos taurus	9913	SAMN05788536	PRJNA343262	Hereford	non-African	536103634	99.6	16.5
Bos taurus	9913	SAMN05788537	PRJNA343262	Hereford	non-African	414675188	99.6	15.2
Bos taurus	9913	SAMN05788538	PRJNA343262	Hereford	non-African	437826602	99.6	15.2
Bos taurus	9913	SAMN05788539	PRJNA343262	Hereford	non-African	427282376	99.6	15.9
Bos taurus	9913	SAMN05788540	PRJNA343262	Hereford	non-African	443062246	99.6	16.5
Bos taurus	9913	SAMN05948863	PRJNA343262	Angus	non-African	532007014	99.6	16.9
Bos javanicus	9906	SAMN08323868	PRJNA427536	Bos javanicus	Bos javanicus	465312132	99	20.1
Bos taurus	9913	SAMN10940449	PRJNA343262	Angus	non-African	391481558	99.4	15.6
Bos taurus	9913	SAMN10940477	PRJNA343262	Angus	non-African	480079824	99.7	17.8
Bos taurus	9913	SAMN10940478	PRJNA343262	Angus	non-African	447578506	99.6	16.8
Bos taurus	9913	SAMN10940479	PRJNA343262	Angus	non-African	430244266	99.6	16.3
Bos taurus	9913	SAMN10940545	PRJNA343262	Limosine	non-African	769252820	99.7	29.6
Bos taurus	9913	SAMN10940546	PRJNA343262	Charolais	non-African	386442030	99.6	14.5
Bos taurus	9913	SAMN10940547	PRJNA343262	Charolais	non-African	386673472	99.6	14.8
Bos taurus	9913	SAMN10940548	PRJNA343262	Charolais	non-African	405306978	99.6	14.9
Bos taurus	9913	SAMN10940549	PRJNA343262	Charolais	non-African	131365740	97	4.9
Bos taurus	9913	SAMN10940550	PRJNA343262	Charolais	non-African	369418834	99.4	12.6
Bos taurus	9913	SAMN10940552	PRJNA343262	Charolais	non-African	424359594	99.6	15.9
Bos taurus	9913	SAMN10940554	PRJNA343262	Charolais	non-African	384634036	99.6	14.6
Bos taurus	9913	SAMN10940556	PRJNA343262	Charolais	non-African	419231200	99.6	15.7
Bos taurus	9913	SAMN10940571	PRJNA343262	Maine Anjou	non-African	484066216	99.6	27.2
Bos taurus	9913	SAMN10940573	PRJNA343262	Maine Anjou	non-African	251643508	99.5	13.4

Bos taurus	9913	SAMN10940577	PRJNA343262	Maine Anjou	non-African	526655092	99.6	29.5
Bos taurus	9913	SAMN10940578	PRJNA343262	Maine Anjou	non-African	500899832	99.6	28.1
Bos taurus	9913	SAMN10940579	PRJNA343262	Maine Anjou	non-African	477082556	99.6	26.8
Bos taurus	9913	SAMN10940698	PRJNA343262	Jersey	non-African	322967122	99.5	18.2
Bos taurus	9913	SAMN13655857	PRJNA343262	Salers	non-African	599330328	99.6	33.5
Bos taurus	9913	SAMN13655858	PRJNA343262	Salers	non-African	540458818	99.6	29.9
Bos taurus	9913	SAMN13655859	PRJNA343262	Salers	non-African	575673150	99.6	32.6
Bos taurus	9913	SAMN13655860	PRJNA343262	Salers	non-African	548589486	99.6	30.1
Bos taurus	9913	SAMN13655861	PRJNA343262	Salers	non-African	568862178	99.6	30.1
Bos taurus	9913	SAMN13655863	PRJNA343262	Salers	non-African	577574384	99.6	31.4
Bos taurus	9913	SAMN13655864	PRJNA343262	Salers	non-African	611283998	99.7	34.0
Bos taurus	9913	SAMN13655865	PRJNA343262	Salers	non-African	581185784	99.6	31.8
Bos taurus	9913	SAMN13655866	PRJNA343262	Shorthorn	non-African	604253394	99.6	33.2
Bos taurus	9913	SAMN13655867	PRJNA343262	Shorthorn	non-African	500922426	99.6	27.1
Bos taurus	9913	SAMN13655870	PRJNA343262	Shorthorn	non-African	620489052	99.6	34.7
Bos taurus	9913	SAMN13655875	PRJNA343262	Shorthorn	non-African	583880724	99.6	31.9
Bos taurus	9913	SAMN13655876	PRJNA343262	Shorthorn	non-African	601001868	99.6	31.1
Bos taurus	9913	SAMN13655877	PRJNA343262	Shorthorn	non-African	575662130	99.6	32.1
Bos taurus	9913	SAMN13655878	PRJNA343262	Shorthorn	non-African	652708620	99.6	35.7
Bos taurus	9913	SAMN13655879	PRJNA343262	Shorthorn	non-African	625741032	99.6	35.5
Bos taurus	9913	SAMN13655880	PRJNA343262	Shorthorn	non-African	610420370	99.6	31.9
Bos taurus	9913	SAMN13655881	PRJNA343262	Shorthorn	non-African	561345378	99.6	30.0
Bos taurus	9913	SAMN13655882	PRJNA343262	Shorthorn	non-African	563134218	99.6	29.7
Bos taurus	9913	SAMN13655884	PRJNA343262	Shorthorn	non-African	592230846	99.6	32.0
Bos taurus	9913	SAMN13655885	PRJNA343262	Shorthorn	non-African	612053492	99.6	34.2
Bos taurus	9913	SAMN15514477	PRJNA574857	Arsi	African	299570902	99.3	11.4
Bos taurus	9913	SAMN15514478	PRJNA574857	Arsi	African	319381534	99.4	12.1
Bos taurus	9913	SAMN15514479	PRJNA574857	Arsi	African	341155266	99.5	13.0

Bos taurus	9913	SAMN15514480	PRJNA574857	Arsi	African	327882872	99.5	12.3
Bos taurus	9913	SAMN15514481	PRJNA574857	Arsi	African	319332866	99.5	12.2
Bos taurus	9913	SAMN15514482	PRJNA574857	Arsi	African	310548578	99.5	11.7
Bos taurus	9913	SAMN15514483	PRJNA574857	Arsi	African	338190090	99.3	12.8
Bos taurus	9913	SAMN15514484	PRJNA574857	Arsi	African	313996070	99.4	11.9
Bos taurus	9913	SAMN15514485	PRJNA574857	Arsi	African	301841794	99.4	11.3
Bos taurus	9913	SAMN15514486	PRJNA574857	Arsi	African	334182524	99.4	12.7
Bos taurus	9913	SAMN15514487	PRJNA574857	Barka	African	296465992	99.5	11.2
Bos taurus	9913	SAMN15514488	PRJNA574857	Barka	African	325351690	99.4	12.4
Bos taurus	9913	SAMN15514490	PRJNA574857	Barka	African	314005698	99.4	11.9
Bos taurus	9913	SAMN15514491	PRJNA574857	Barka	African	325063740	99.5	12.3
Bos taurus	9913	SAMN15514492	PRJNA574857	Barka	African	337876956	99.4	12.8
Bos taurus	9913	SAMN15514493	PRJNA574857	Barka	African	297704192	99.4	11.6
Bos taurus	9913	SAMN15514494	PRJNA574857	Barka	African	322269136	99.3	12.3
Bos taurus	9913	SAMN15514495	PRJNA574857	Barka	African	359411990	99.5	13.5
Bos taurus	9913	SAMN15514497	PRJNA574857	Butana	African	297803452	99.4	11.2
Bos taurus	9913	SAMN15514498	PRJNA574857	Butana	African	299196434	99.4	11.3
Bos taurus	9913	SAMN15514499	PRJNA574857	Butana	African	297639482	99.5	11.7
Bos taurus	9913	SAMN15514500	PRJNA574857	Butana	African	306826372	99.4	11.7
Bos taurus	9913	SAMN15514501	PRJNA574857	Butana	African	298365598	99.4	11.3
Bos taurus	9913	SAMN15514502	PRJNA574857	Butana	African	348153814	99.5	13.2
Bos taurus	9913	SAMN15514503	PRJNA574857	Butana	African	320262184	99.4	12.0
Bos taurus	9913	SAMN15514504	PRJNA574857	Butana	African	304478980	99.4	11.5
Bos taurus	9913	SAMN15514505	PRJNA574857	Butana	African	313519182	99.4	11.8
Bos taurus	9913	SAMN15514506	PRJNA574857	Butana	African	318021936	99.4	12.1
Bos taurus	9913	SAMN15514507	PRJNA574857	Butana	African	328053600	99.4	12.6
Bos taurus	9913	SAMN15514508	PRJNA574857	Butana	African	299048726	99.4	11.3
Bos taurus	9913	SAMN15514509	PRJNA574857	Butana	African	334041346	99.4	12.8

Bos taurus	9913	SAMN15514510	PRJNA574857	Butana	African	330708206	99.2	12.6
Bos taurus	9913	SAMN15514511	PRJNA574857	Butana	African	345067586	99.4	13.3
Bos taurus	9913	SAMN15514512	PRJNA574857	Butana	African	342148668	99.4	13.0
Bos taurus	9913	SAMN15514513	PRJNA574857	Butana	African	297708252	99.5	11.2
Bos taurus	9913	SAMN15514514	PRJNA574857	Butana	African	301786050	99.4	11.1
Bos taurus	9913	SAMN15514515	PRJNA574857	Butana	African	300887844	99.5	11.1
Bos taurus	9913	SAMN15514516	PRJNA574857	EthiopianBoran	African	335743518	99.4	12.8
Bos taurus	9913	SAMN15514517	PRJNA574857	EthiopianBoran	African	318400460	99.4	12.0
Bos taurus	9913	SAMN15514518	PRJNA574857	EthiopianBoran	African	317133770	99.4	12.1
Bos taurus	9913	SAMN15514519	PRJNA574857	EthiopianBoran	African	329308970	99.4	12.6
Bos taurus	9913	SAMN15514520	PRJNA574857	EthiopianBoran	African	328211686	99.5	12.3
Bos taurus	9913	SAMN15514521	PRJNA574857	EthiopianBoran	African	309114930	99.4	11.5
Bos taurus	9913	SAMN15514522	PRJNA574857	EthiopianBoran	African	302166786	99.4	11.4
Bos taurus	9913	SAMN15514523	PRJNA574857	EthiopianBoran	African	310348566	99.4	11.6
Bos taurus	9913	SAMN15514524	PRJNA574857	EthiopianBoran	African	319337224	99.4	12.0
Bos taurus	9913	SAMN15514525	PRJNA574857	EthiopianBoran	African	318372208	99.4	12.1
Bos taurus	9913	SAMN15514526	PRJNA574857	Goffa	African	326253396	99.4	12.6
Bos taurus	9913	SAMN15514527	PRJNA574857	Goffa	African	331064596	99.4	12.6
Bos taurus	9913	SAMN15514528	PRJNA574857	Goffa	African	320548404	99.5	12.3
Bos taurus	9913	SAMN15514529	PRJNA574857	Goffa	African	266843272	99.4	10.4
Bos taurus	9913	SAMN15514530	PRJNA574857	Goffa	African	304852762	99.5	12.1
Bos taurus	9913	SAMN15514531	PRJNA574857	Goffa	African	310385168	99.4	11.8
Bos taurus	9913	SAMN15514532	PRJNA574857	Goffa	African	336685622	99.4	12.8
Bos taurus	9913	SAMN15514533	PRJNA574857	Goffa	African	318056964	99.4	12.1
Bos taurus	9913	SAMN15514534	PRJNA574857	Goffa	African	250576934	99.3	9.4
Bos taurus	9913	SAMN15514535	PRJNA574857	Goffa	African	285562132	99.4	10.8
Bos taurus	9913	SAMN15514536	PRJNA574857	Kenana	African	297669986	99.3	11.1
Bos taurus	9913	SAMN15514537	PRJNA574857	Kenana	African	310283640	99.4	11.7

Bos taurus	9913	SAMN15514538	PRJNA574857	Kenana	African	311170654	99.4	11.8
Bos taurus	9913	SAMN15514539	PRJNA574857	Kenana	African	340139634	99.5	13.1
Bos taurus	9913	SAMN15514540	PRJNA574857	Mursi	African	318777690	99.5	12.2
Bos taurus	9913	SAMN15514541	PRJNA574857	Mursi	African	298606306	99.4	11.2
Bos taurus	9913	SAMN15514542	PRJNA574857	Mursi	African	318097542	99.4	12.2
Bos taurus	9913	SAMN15514543	PRJNA574857	Mursi	African	348125120	99.3	13.3
Bos taurus	9913	SAMN15514544	PRJNA574857	Mursi	African	293755244	99.5	11.0
Bos taurus	9913	SAMN15514545	PRJNA574857	Mursi	African	291853754	99.5	11.1
Bos taurus	9913	SAMN15514546	PRJNA574857	Mursi	African	287980868	99.5	10.9
Bos taurus	9913	SAMN15514547	PRJNA574857	Mursi	African	239023462	99.3	9.1
Bos taurus	9913	SAMN15514548	PRJNA574857	Mursi	African	313780012	99.4	12.1
Bos taurus	9913	SAMN15514549	PRJNA574857	Mursi	African	312742686	99.4	12.0
Bos taurus	9913	SAMN15514550	PRJNA574857	Afar	African	344316232	99.4	13.2
Bos taurus	9913	SAMN15514551	PRJNA574857	Afar	African	311136836	99.4	11.9
Bos taurus	9913	SAMN15514552	PRJNA574857	Afar	African	322607306	99.4	12.2
Bos taurus	9913	SAMN15514553	PRJNA574857	Afar	African	312247322	99.5	11.9
Bos taurus	9913	SAMN15514554	PRJNA574857	Afar	African	323482306	99.3	12.4
Bos taurus	9913	SAMN15514555	PRJNA574857	Afar	African	302469154	99.5	11.5
Bos taurus	9913	SAMN15514556	PRJNA574857	Afar	African	316782804	99.5	12.1
Bos taurus	9913	SAMN15514557	PRJNA574857	Afar	African	303937638	99.4	11.4
Bos taurus	9913	SAMN15514558	PRJNA574857	Afar	African	320264568	99.4	12.1
Bos taurus	9913	SAMN15514559	PRJNA574857	Ndama	African	322460204	99.4	12.4
Bos taurus	9913	SAMN15514560	PRJNA574857	Ndama	African	316699934	99.5	12.1
Bos taurus	9913	SAMN15514561	PRJNA574857	Ndama	African	313233898	99.5	12.0
Bos taurus	9913	SAMN15514562	PRJNA574857	Sheko	African	309494758	99.4	12.0
Bos taurus	9913	SAMN15514563	PRJNA574857	Sheko	African	362917276	99.5	14.1
Bos taurus	9913	SAMN15514564	PRJNA574857	Sheko	African	335854442	99.5	13.0
Bos taurus	9913	SAMN15514565	PRJNA574857	Sheko	African	310020578	99.5	12.0

Bos taurus	9913	SAMN15514566	PRJNA574857	Sheko	African	325129958	99.4	12.1
Bos taurus	9913	SAMN15514567	PRJNA574857	Sheko	African	328865892	99.5	12.8
Bos taurus	9913	SAMN15514568	PRJNA574857	Sheko	African	322812378	99.5	12.4
Bos taurus	9913	SAMN15514569	PRJNA574857	Sheko	African	300092484	99.4	11.7
Bos taurus	9913	SAMN15514570	PRJNA574857	Sheko	African	326314302	99.5	12.7
Bos taurus	9913	SAMN15514571	PRJNA574857	Fogera	African	318183816	99.4	12.4
Bos taurus	9913	SAMN15514572	PRJNA574857	Fogera	African	306232394	99.5	11.9
Bos taurus	9913	SAMN15514573	PRJNA574857	Fogera	African	297766326	99.3	11.2
Bos taurus	9913	SAMN15514574	PRJNA574857	Fogera	African	307298294	99.4	12.0
Bos taurus	9913	SAMN15514575	PRJNA574857	Fogera	African	338750828	99.5	13.2
Bos taurus	9913	SAMN15514576	PRJNA574857	Fogera	African	356042402	99.5	13.7
Bos taurus	9913	SAMN15514577	PRJNA574857	Fogera	African	315100798	99.4	12.3
Bos taurus	9913	SAMN15514578	PRJNA574857	Fogera	African	300754936	99.4	11.2
Bos taurus	9913	SAMN15514579	PRJNA574857	Fogera	African	323192094	99.4	12.6
Bos taurus	9913	SAMN15514580	PRJNA574857	Horro	African	248690244	99.4	9.4
Bos taurus	9913	SAMN15514581	PRJNA574857	Horro	African	301990200	99.5	11.4
Bos taurus	9913	SAMN15514582	PRJNA574857	Horro	African	326108458	99.4	12.4
Bos taurus	9913	SAMN15514583	PRJNA574857	Horro	African	267521506	99.4	10.1
Bos taurus	9913	SAMN15514584	PRJNA574857	Horro	African	291476430	99.4	10.9
Bos taurus	9913	SAMN15514585	PRJNA574857	Horro	African	263203788	99.4	10.0
Bos taurus	9913	SAMN15514586	PRJNA574857	Horro	African	308091598	99.5	11.7
Bos taurus	9913	SAMN15514587	PRJNA574857	Horro	African	297930266	99.4	11.3
Bos taurus	9913	SAMN15514588	PRJNA574857	Horro	African	251584502	99.3	9.6
Bos taurus	9913	SAMN15514589	PRJNA574857	Horro	African	310461514	99.4	11.7
Bos taurus	9913	SAMN15514590	PRJNA574857	Horro	African	316095718	99.4	12.0

Table S2. Genes involved in coronavirus infection investigated in the present study.

Gene name	Gene symbol	Cattle				Human
		BTA location[1]	Gene[2]	Transcript[3]	Protein - Length[4]	Protein[5]
Angiotensin I converting enzyme 2	*ACE2*	X:127901491-127951491	ENSBTAG00000034402	ENSBTAT00000048730.4	A0A452DJE0	Q9BYF1
Alanyl aminopeptidase, membrane	*ANPEP*	21:21195680-21213021	ENSBTAG00000016881	ENSBTAT00000068383.1	A0A3Q1MB09	P15144
CEA Cell Adhesion Molecule 1	*CEACAM1*	18:50817523-50837100	ENSBTAG00000005615	ENSBTAT00000069303.1	A0A3Q1MQ27	P13688
Dipeptidyl peptidase 4	*DPP4*	2:34382575-34466656	ENSBTAG00000048246	ENSBTAT00000056886.3.1	P81425	P27487
Furin, Paired Basic Amino Acid Cleaving Enzyme	*FURIN*	21:21780694-21793171	ENSBTAG00000002939	ENSBTAT00000072776.1	B0JYR0	P09958
Transmembrane serine protease 2	*TMPRSS2*	1:141713171-141747814	ENSBTAG00000009132	ENSBTAT00000012036.5.2	A2VDV7	O15393

[1] Bovine chromosome, starting position, ending position. Genomic coordinates are based on the ARS-UCD1.2 reference genome.

[2] Ensembl gene identifier.

[3] Ensembl canonical transcript identifier (it is defined as the longest CCDS translation with no stop codons).

[4] UniProtKB accession number related to the Ensembl canonical transcript. The number of residues of the protein is reported.

[5] UniProtKB accession number.

animals

Table S3. Variants (n. 82) identified in at least one *B. taurus* population.

Gene	dbSNP	SAP	SAP	SIFT	Allele freq. *Bos* genus	Class	Angus	Blonde d'Aquitaine	Brown Swiss	Charolais	Chianina	Gelbvieh	Hereford	Holstein	Jersey	Limosine	Maine Anjou	Original Braunvieh	Piedmontese	Red Angus	Romagnola	Salers	Shorthorn	Simmental	Tyrolean Grey	Afar	Arsi	Barka	Butana	Ethiopian Boran	Fogera	Goffa	Horro	Kenana	Mursi	N'Dama	Sheko	*B. t. indicus*	*B. grunniens*	*B. javanicus*
						Origin	non-African																			African												Other		
						N. animals	28	1	8	29	3	25	33	121	14	6	12	10	8	10	7	8	13	42	8	9	10	8	19	10	9	10	11	4	10	3	9	1	3	1
ACE2	rs482846303	18	M18I	tolerated(0.26)	0,006	Private SAP	0,000	0,000	0,000	0,000	0,000	0,000	0,000	0,000	0,250	0,000	0,000	0,000	0,000	0,000	0,000	0,000	0,000	0,000	0,000	0,000	0,000	0,000	0,000	0,000	0,000	0,000	0,000	0,000	0,000	0,000	0,000	0,000	0,000	0,000
ACE2	-	105	G105S	tolerated(0.13)	0,001	Private SAP	0,000	NA	0,000	0,000	0,000	0,000	0,000	0,000	0,000	0,000	0,000	0,000	0,000	0,000	0,000	0,000	0,000	0,000	0,000	0,000	0,000	0,000	0,026	0,000	0,000	0,000	0,000	0,000	0,000	0,000	0,000	0,000	0,000	0,000
ACE2	-	114	L114HV	-	0,001	Private SAP	0,024	0,000	0,000	0,000	0,000	0,000	0,000	0,000	0,000	0,000	0,000	0,000	0,000	0,000	0,000	0,000	0,000	0,000	0,000	0,000	0,000	0,000	0,000	0,000	0,000	0,000	0,000	0,000	0,000	0,000	0,000	0,000	0,000	0,000
ACE2	-	114	L114LGSAYIGLSRNK	-	0,001	Private SAP	0,024	0,000	0,000	0,000	0,000	0,000	0,000	0,000	0,000	0,000	0,000	0,000	0,000	0,000	0,000	0,000	0,000	0,000	0,000	0,000	0,000	0,000	0,000	0,000	0,000	0,000	0,000	0,000	0,000	0,000	0,000	0,000	0,000	0,000
ACE2	rs715723143	135	E135K	tolerated(0.68)	0,014	non-private SAP	0,180	0,000	0,000	0,000	0,000	0,000	0,000	0,000	0,000	0,000	0,091	0,050	0,000	0,000	0,000	0,000	0,000	0,000	0,125	0,000	0,000	0,000	0,000	0,000	0,000	0,000	0,000	0,000	0,000	0,000	0,000	0,000	0,000	0,000
ACE2	-	325	A325V	tolerated(0.15)	0,005	non-private SAP	0,000	NA	0,000	0,000	0,000	0,000	0,000	0,000	0,000	0,000	0,000	0,000	0,000	0,000	0,000	0,000	0,000	0,000	0,000	0,000	0,056	0,000	0,079	0,000	0,000	0,000	0,050	0,000	0,000	0,000	0,000	0,000	0,000	0,000
ACE2	-	591	K591Q	tolerated_low_confidence(0.64)	0,001	Private SAP	0,000	0,000	0,000	0,000	0,000	0,000	0,000	0,000	0,000	0,000	0,000	0,000	0,000	0,000	0,000	0,000	0,000	0,000	0,000	0,000	0,000	0,000	0,000	0,000	0,056	0,000	0,000	0,000	0,000	0,000	0,000	0,000	0,000	0,000
ACE2	rs526864493	595	E595A	tolerated_low_confidence(0.07)	0,059	non-private SAP	0,000	0,000	0,000	0,000	0,000	0,000	0,000	0,000	0,000	0,000	0,000	0,000	0,000	0,000	0,000	0,000	0,000	0,000	0,000	0,333	0,250	0,563	0,278	0,200	0,111	0,400	0,300	0,000	0,056	0,000	0,111	0,500	0,000	1,000
ACE2	-	597	R597K	tolerated_low_confidence(1)	0,001	Private SAP	0,000	0,000	0,000	0,000	0,000	0,000	0,000	0,000	0,000	0,000	0,000	0,000	0,000	0,000	0,000	0,000	0,000	0,000	0,000	0,000	0,000	0,000	0,000	0,000	0,056	0,000	0,000	0,000	0,000	0,000	0,000	0,000	0,000	0,000
ACE2	rs463788353	601	V601I	tolerated_low_confidence(0.85)	0,058	non-private SAP	0,000	0,000	0,000	0,000	0,000	0,000	0,000	0,000	0,000	0,000	0,000	0,000	0,000	0,000	0,000	0,000	0,000	0,000	0,000	0,333	0,350	0,563	0,263	0,125	0,111	0,400	0,333	0,000	0,000	0,000	0,111	0,500	0,000	0,000
ANPEP	rs715733499	20	V20M	deleterious(0.03)	0,018	non-private SAP	0,000	NA	0,000	0,017	0,000	0,000	0,000	0,000	0,000	0,000	0,000	0,000	0,000	0,000	0,000	0,000	0,000	0,000	0,000	0,200	0,125	0,000	0,132	0,000	0,071	0,000	0,000	0,000	0,000	0,000	0,000	0,000	0,000	0,500
ANPEP	rs1115122772	46	A46V	tolerated(0.55)	0,023	non-private SAP	0,000	0,000	0,000	0,000	0,000	0,000	0,000	0,000	0,000	0,000	0,000	0,000	0,000	0,000	0,000	0,000	0,000	0,000	0,000	0,143	0,143	0,000	0,083	0,063	0,188	0,063	0,000	0,000	0,286	0,500	0,083	0,000	0,000	0,000
ANPEP	-	79	P79X	-	0,001	Private SAP	0,000	0,000	0,000	0,000	0,000	0,000	0,000	0,000	0,036	0,000	0,000	0,000	0,000	0,000	0,000	0,000	0,000	0,000	0,000	0,000	0,000	0,000	0,000	0,000	0,000	0,000	0,000	0,000	0,000	0,000	0,000	0,000	0,000	0,000
ANPEP	rs41970582	135	V135A	tolerated(0.78)	0,708	non-private SAP	0,769	1,000	0,643	0,750	1,000	0,762	0,783	0,636	0,964	0,667	0,625	0,600	0,563	0,833	0,571	0,688	0,808	0,467	0,938	0,944	0,944	0,813	0,875	0,857	0,700	0,875	0,818	0,750	0,778	1,000	0,786	1,000	0,000	1,000
ANPEP	-	144	A144P	tolerated(0.36)	0,001	Private SAP	0,000	0,000	0,000	0,000	0,000	0,000	0,000	0,000	0,000	0,000	0,000	0,000	0,000	0,000	0,000	0,000	0,000	0,000	0,000	0,000	0,000	0,000	0,000	0,000	0,000	0,000	0,000	0,000	0,056	0,000	0,000	0,000	0,000	0,000
ANPEP	-	146	E146D	tolerated(0.54)	0,001	Private SAP	0,000	0,000	0,000	0,000	0,000	0,000	0,000	0,000	0,000	0,000	0,000	0,000	0,000	0,000	0,000	0,000	0,000	0,000	0,000	0,000	0,000	0,000	0,000	0,000	0,000	0,000	0,000	0,000	0,056	0,000	0,000	0,000	0,000	0,000
ANPEP	-	149	R149K	tolerated(0.76)	0,001	Private SAP	0,000	0,000	0,000	0,000	0,000	0,000	0,000	0,000	0,000	0,000	0,000	0,000	0,000	0,000	0,000	0,000	0,000	0,000	0,000	0,000	0,000	0,000	0,000	0,000	0,000	0,000	0,000	0,000	0,056	0,000	0,000	0,000	0,000	0,000
ANPEP	-	170	G70X	-	0,001	Private SAP	0,000	0,000	0,000	0,000	0,000	0,000	0,000	0,000	0,000	0,000	0,000	0,000	0,000	0,000	0,000	0,000	0,000	0,000	0,000	0,000	0,063	0,000	0,000	0,000	0,000	0,000	0,000	0,000	0,000	0,000	0,000	0,000	0,000	0,000
ANPEP	rs800590783	185	D185N	deleterious(0.02)	0,003	non-private SAP	0,000	0,000	0,000	0,036	0,000	0,000	0,000	0,000	0,000	0,000	0,000	0,000	0,071	0,000	0,000	0,000	0,000	0,000	0,000	0,000	0,000	0,000	0,000	0,000	0,000	0,000	0,000	0,000	0,000	0,000	0,000	0,000	0,000	0,000
ANPEP	-	229	T229K	tolerated(0.29)	0,002	non-private SAP	0,000	0,000	0,000	0,000	0,000	0,000	0,000	0,000	0,000	0,000	0,000	0,000	0,000	0,000	0,000	0,000	0,000	0,000	0,000	0,000	0,000	0,000	0,026	0,000	0,056	0,000	0,000	0,000	0,000	0,000	0,000	0,000	0,000	0,000
ANPEP	-	229	T229A	tolerated(0.34)	0,002	non-private SAP	0,000	0,000	0,000	0,000	0,000	0,000	0,000	0,000	0,000	0,000	0,000	0,000	0,000	0,000	0,000	0,000	0,000	0,000	0,000	0,000	0,000	0,000	0,026	0,000	0,056	0,000	0,000	0,000	0,000	0,000	0,000	0,000	0,000	0,000
ANPEP	-	247	P247L	tolerated(0.36)	0,001	Private SAP	0,000	0,000	0,000	0,000	0,000	0,000	0,000	0,000	0,000	0,000	0,000	0,000	0,000	0,000	0,000	0,000	0,000	0,000	0,000	0,000	0,000	0,000	0,000	0,000	0,056	0,000	0,000	0,000	0,000	0,000	0,000	0,000	0,000	0,000
ANPEP	rs210492311	257	G257R	tolerated(0.24)	0,004	non-private SAP	0,000	0,000	0,000	0,000	0,000	0,000	0,000	0,000	0,000	0,000	0,000	0,000	0,000	0,000	0,000	0,000	0,000	0,017	0,000	0,000	0,000	0,000	0,000	0,056	0,000	0,056	0,050	0,000	0,000	0,000	0,000	0,000	0,000	0,000
ANPEP	-	260	N260D	tolerated(1)	0,001	Private SAP	0,000	0,000	0,000	0,000	0,000	0,000	0,000	0,000	0,000	0,000	0,000	0,000	0,000	0,000	0,000	0,000	0,000	0,000	0,000	0,000	0,000	0,000	0,000	0,000	0,000	0,000	0,050	0,000	0,000	0,000	0,000	0,000	0,000	0,000
ANPEP	-	363	A363S	tolerated(0.45)	0,001	Private SAP	0,000	0,000	0,000	0,000	0,000	0,000	0,000	0,000	0,000	0,000	0,000	0,000	0,000	0,000	0,000	0,000	0,000	0,000	0,000	0,000	0,000	0,071	0,000	0,000	0,000	0,000	0,000	0,000	0,000	0,000	0,000	0,000	0,000	0,000
ANPEP	-	398	L398M	tolerated(0.61)	0,001	Private SAP	0,000	0,000	0,000	0,000	0,000	0,000	0,000	0,000	0,000	0,000	0,000	0,000	0,000	0,000	0,000	0,000	0,000	0,000	0,000	0,000	0,000	0,000	0,028	0,000	0,000	0,000	0,000	0,000	0,000	0,000	0,000	0,000	0,000	0,000
ANPEP	-	399	A399D	tolerated(0.21)	0,001	Private SAP	0,000	0,000	0,000	0,000	0,000	0,000	0,000	0,000	0,000	0,000	0,000	0,000	0,000	0,000	0,000	0,000	0,000	0,000	0,000	0,000	0,000	0,000	0,028	0,000	0,000	0,000	0,000	0,000	0,000	0,000	0,000	0,000	0,000	0,000
ANPEP	-	439	R439H	tolerated(0.05)	0,002	Private SAP	0,000	NA	0,000	0,000	0,000	0,000	0,000	0,000	0,000	0,000	0,000	0,000	0,000	0,000	0,000	0,000	0,000	0,000	0,000	0,000	0,000	0,000	0,000	0,000	0,111	0,000	0,000	0,000	0,000	0,000	0,000	0,000	0,000	0,000
ANPEP	-	439	R439C	deleterious(0.01)	0,001	Private SAP	0,000	NA	0,000	0,019	0,000	0,000	0,000	0,000	0,000	0,000	0,000	0,000	0,000	0,000	0,000	0,000	0,000	0,000	0,000	0,000	0,000	0,000	0,000	0,000	0,000	0,000	0,000	0,000	0,000	0,000	0,000	0,000	0,000	0,000
ANPEP	rs714608634	523	E523D	tolerated(1)	0,024	non-private SAP	0,000	0,000	0,000	0,000	0,000	0,000	0,000	0,000	0,000	0,000	0,000	0,000	0,000	0,000	0,000	0,000	0,000	0,000	0,000	0,000	0,000	0,188	0,063	0,000	0,214	0,125	0,063	0,000	0,200	0,000	0,143	0,000	1,000	0,000
ANPEP	rs436687789	559	I559L	tolerated(0.13)	0,002	non-private SAP	0,000	0,000	0,000	0,000	0,000	0,000	0,000	0,000	0,000	0,000	0,000	0,000	0,000	0,000	0,000	0,000	0,000	0,000	0,000	0,056	0,000	0,000	0,000	0,000	0,000	0,000	0,000	0,000	0,000	0,000	0,063	0,000	0,000	0,000
ANPEP	-	562	K562E	tolerated(0.66)	0,002	non-private SAP	0,000	0,000	0,000	0,000	0,000	0,000	0,000	0,000	0,000	0,000	0,000	0,000	0,000	0,000	0,000	0,000	0,000	0,000	0,000	0,056	0,000	0,000	0,000	0,000	0,000	0,000	0,000	0,000	0,000	0,000	0,063	0,000	0,000	0,000
ANPEP	-	581	L581P	tolerated(0.27)	0,001	Private SAP	0,000	0,000	0,000	0,000	0,000	0,000	0,016	0,000	0,000	0,000	0,000	0,000	0,000	0,000	0,000	0,000	0,000	0,000	0,000	0,000	0,000	0,000	0,000	0,000	0,000	0,000	0,000	0,000	0,000	0,000	0,000	0,000	0,000	0,000
ANPEP	rs207782807	649	R649P	tolerated(0.35)	0,195	non-private SAP	0,056	0,000	0,071	0,125	0,000	0,065	0,063	0,171	0,036	0,083	0,125	0,000	0,125	0,050	0,000	0,188	0,000	0,154	0,000	0,625	0,278	0,563	0,737	0,222	0,444	0,222	0,455	0,375	0,611	1,000	0,222	0,500	0,500	1,000
ANPEP	rs208836615	689	M689L	tolerated(0.68)	0,081	non-private SAP	0,077	0,000	0,071	0,096	0,000	0,091	0,061	0,162	0,042	0,083	0,125	0,000	0,125	0,050	0,000	0,188	0,000	0,172	0,000	0,000	0,000	0,000	0,000	0,000	0,000	0,000	0,000	0,000	0,000	0,000	0,000	0,000	0,000	0,000
ANPEP	rs436355025	722	K722R	tolerated(1)	0,086	non-private SAP	0,000	0,000	0,000	0,018	0,000	0,000	0,000	0,000	0,000	0,000	0,000	0,000	0,000	0,000	0,000	0,000	0,000	0,000	0,000	0,389	0,150	0,438	0,579	0,200	0,333	0,150	0,545	0,250	0,450	0,000	0,250	0,500	0,000	1,000
ANPEP	rs467826408	740	T740I	deleterious(0.02)	0,085	non-private SAP	0,000	0,000	0,000	0,017	0,000	0,021	0,000	0,000	0,000	0,000	0,000	0,000	0,000	0,000	0,000	0,000	0,000	0,000	0,000	0,389	0,150	0,500	0,632	0,200	0,333	0,150	0,500	0,250	0,400	0,000	0,222	0,500	0,000	1,000
ANPEP	rs523244588	806	E806A	tolerated(0.65)	0,099	non-private SAP	0,000	0,000	0,000	0,018	0,000	0,000	0,000	0,000	0,000	0,000	0,000	0,000	0,000	0,000	0,000	0,000	0,000	0,000	0,000	0,333	0,214	0,688	0,694	0,222	0,313	0,100	0,550	0,375	0,500	0,000	0,357	0,500	1,000	1,000
ANPEP	rs525829647	813	N813S	tolerated(0.54)	0,092	non-private SAP	0,000	0,000	0,000	0,019	0,000	0,000	0,000	0,000	0,000	0,000	0,000	0,000	0,000	0,000	0,000	0,000	0,000	0,000	0,000	0,313	0,214	0,688	0,711	0,250	0,278	0,150	0,500	0,500	0,500	0,000	0,313	0,500	0,000	0,500
ANPEP	-	879	I879L	tolerated(0.2)	0,001	Private SAP	0,000	NA	0,000	0,000	0,000	0,000	0,000	0,000	0,000	0,000	0,000	0,000	0,000	0,000	0,000	0,000	0,000	0,000	0,000	0,000	0,000	0,000	0,000	0,000	0,000	0,050	0,000	0,000	0,000	0,000	0,000	0,000	0,000	0,000
ANPEP	-	948	N948K	tolerated(1)	0,001	Private SAP	0,000	0,000	0,000	0,000	0,000	0,000	0,000	0,000	0,000	0,000	0,000	0,000	0,000	0,000	0,000	0,000	0,000	0,000	0,000	0,000	0,000	0,000	0,000	0,000	0,000	0,000	0,000	0,000	0,000	0,000	0,056	0,000	0,000	0,000
ANPEP	rs211627711	959	N959H	tolerated(0.06)	0,087	non-private SAP	0,074	0,000	0,071	0,125	0,000	0,068	0,016	0,149	0,036	0,083	0,125	0,000	0,000	0,050	0,000	0,188	0,000	0,000	0,000	0,000	0,056	0,188	0,111	0,050	0,125	0,167	0,227	0,000	0,100	0,000	0,167	0,000	0,000	0,000
ANPEP	rs210982416	963	D963E	tolerated(1)	0,142	non-private SAP	0,000	0,000	0,357	0,000	0,000	0,000	0,000	0,000	0,000	0,000	0,000	0,000	0,000	0,000	0,000	0,000	0,000	0,015	0,125	0,722	0,611	0,500	0,853	0,556	0,563	0,389	0,500	0,500	0,700	0,667	0,333	0,500	0,000	0,500
ANPEP	rs1116627945	963	D963H	deleterious(0.02)	0,004	non-private SAP	0,000	0,000	0,000	0,000	0,000	0,000	0,000	0,000	0,000	0,000	0,000	0,000	0,000	0,000	0,000	0,000	0,000	0,000	0,000	0,063	0,056	0,000	0,000	0,000	0,000	0,000	0,000	0,000	0,000	0,333	0,000	0,000	0,000	0,000
ANPEP	-	164-165	KS/X	-	0,001	Private SAP	0,000	0,000	0,000	0,000	0,000	0,000	0,000	0,000	0,000	0,000	0,000	0,000	0,000	0,000	0,000	0,000	0,000	0,000	0,000	0,000	0,071	0,000	0,000	0,000	0,000	0,000	0,000	0,000	0,000	0,000	0,000	0,000	0,000	0,000
ANPEP	-	166-168	SLE/SX	-	0,001	Private SAP	0,000	0,000	0,000	0,000	0,000	0,000	0,000	0,000	0,000	0,000	0,000	0,000	0,000	0,000	0,000	0,000	0,000	0,000	0,000	0,000	0,071	0,000	0,000	0,000	0,000	0,000	0,000	0,000	0,000	0,000	0,000	0,000	0,000	0,000
ANPEP	-	172-173	-/A	-	0,001	Private SAP	0,000	0,000	0,000	0,000	0,000	0,000	0,000	0,000	0,000	0,000	0,000	0,000	0,000	0,000	0,000	0,000	0,000	0,000	0,000	0,000	0,071	0,000	0,000	0,000	0,000	0,000	0,000	0,000	0,000	0,000	0,000	0,000	0,000	0,000
CEACAM1	rs799779883	13	I13T	deleterious(0.04)	0,005	non-private SAP	0,000	0,000	0,000	0,000	0,000	0,000	0,000	0,004	0,000	0,000	0,083	0,000	0,000	0,000	0,000	0,000	0,077	0,000	0,000	0,000	0,000	0,000	0,000	0,000	0,000	0,000	0,000	0,000	0,000	0,000	0,000	0,000	0,000	0,000
CEACAM1	-	36	L36P	tolerated(0.07)	0,068	non-private SAP	0,000	0,000	0,000	0,000	0,000	0,000	0,000	0,000	0,000	0,000	0,000	0,000	0,000	0,000	0,000	0,000	0,000	0,000	0,000	0,286	0,286	0,375	0,188	0,063	0,357	0,556	0,150	0,250	0,278	0,000	0,333	0,000	0,833	0,500
CEACAM1	-	38	I38T	tolerated(0.07)	0,020	non-private SAP	0,018	0,000	0,063	0,036	0,000	0,020	0,061	0,013	0,000	0,000	0,042	0,000	0,000	0,000	0,071	0,000	0,000	0,013	0,000	0,000	0,071	0,000	0,031	0,000	0,000	0,000	0,100	0,000	0,000	0,000	0,000	0,000	0,000	0,000
CEACAM1	rs525779095	40	T40M	deleterious(0)	0,061	non-private SAP	0,000	0,000	0,000	0,000	0,000	0,000	0,000	0,000	0,000	0,000	0,000	0,000	0,000	0,000	0,000	0,000	0,000	0,000	0,000	0,375	0,214	0,357	0,222	0,063	0,313	0,556	0,200	0,250	0,278	0,000	0,313	0,000	0,000	0,000
CEACAM1	-	41	V41M	tolerated(0.14)	0,020	non-private SAP	0,018	0,500	0,000	0,034	0,000	0,020	0,063	0,017	0,000	0,000	0,000	0,050	0,000	0,050	0,143	0,000	0,038	0,013	0,000	0,000	0,000	0,000	0,000	0,000	0,000	0,000	0,000	0,000	0,000	0,000	0,000	0,000	0,167	0,000
CEACAM1	-	49	S/*	-	0,001	Private SAP	0,000	0,000	0,000	0,000	0,000	0,000	0,000	0,000	0,000	0,000	0,000	0,000	0,000	0,050	0,000	0,000	0,000	0,000	0,000	0,000	0,000	0,000	0,000	0,000	0,000	0,000	0,000	0,000	0,000	0,000	0,000	0,000	0,000	0,000
CEACAM1	-	50	D50H	deleterious(0)	0,001	Private SAP	0,000	0,000	0,000	0,000	0,000	0,000	0,000	0,000	0,000	0,000	0,000	0,000	0,000	0,056	0,000	0,000	0,000	0,000	0,000	0,000	0,000	0,000	0,000	0,000	0,000	0,000	0,000	0,000	0,000	0,000	0,000	0,000	0,000	0,000
CEACAM1	-	51	V51VN	-	0,001	Private SAP	0,000	0,000	0,000	0,000	0,000	0,000	0,000	0,000	0,000	0,000	0,000	0,000	0,000	0,056	0,000	0,000	0,000	0,000	0,000	0,000	0,000	0,000	0,000	0,000	0,000	0,000	0,000	0,000	0,000	0,000	0,000	0,000	0,000	0,000
CEACAM1	rs41887900	56	H56Q	deleterious(0.03)	0,203	non-private SAP	0,077	0,000	0,063	0,315	0,000	0,196	0,224	0,207	0,167	0,333	0,292	0,250	0,250	0,111	0,000	0,125	0,308	0,143	0,188	0,083	0,214	0,083	0,250	0,375	0,000	0,250	0,333	0,250	0,214	0,000	0,563	0,000	0,000	0,000
CEACAM1	-	58	V58L	tolerated(1)	0,016	non-private SAP	0,000	0,000	0,000	0,000	0,000	0,000	0,000	0,035	0,000	0,000	0,083	0,100	0,000	0,000	0,214	0,000	0,038	0,000	0,000	0,000	0,000	0,000	0,000	0,000	0,000	0,000	0,000	0,000	0,000	0,000	0,000	0,000	0,000	0,000
CEACAM1	rs470684343	59	T59A	deleterious(0.03)	0,015	non-private SAP	0,019	0,000	0,063	0,018	0,000	0,000	0,034	0,022	0,000	0,000	0,000	0,050	0,000	0,000	0,071	0,000	0,000	0,014	0,000	0,000	0,000	0,000	0,000	0,050	0,000	0,000	0,056	0,000	0,000	0,000	0,000	0,000	0,000	0,000
CEACAM1	rs439368541	60	K60N	tolerated(0.12)	0,014	non-private SAP	0,019	0,000	0,063	0,018	0,000	0,000	0,036	0,022	0,000	0,000	0,000	0,050	0,000	0,000	0,071	0,000	0,000	0,014	0,000	0,000	0,000	0,000	0,000	0,000	0,000	0,000	0,056	0,000	0,000	0,000	0,000	0,000	0,000	0,000
CEACAM1	rs41887901	61	N61S	tolerated(0.06)	0,211	non-private SAP	0,100	0,000	0,063	0,340	0,000	0,205	0,241	0,220	0,115	0,333	0,292	0,250	0,214	0,111	0,000	0,125	0,308	0,153	0,188	0,083	0,188	0,071	0,250	0,500	0,000	0,300	0,313	0,250	0,214	0,000	0,556	0,000	0,000	0,000
CEACAM1	-	64	G64A	tolerated(0.17)	0,023	non-private SAP	0,000	0,000	0,000	0,017	0,000	0,025	0,000	0,043	0,000	0,000	0,042	0,100	0,000	0,000	0,286	0,063	0,038	0,000	0,000	0,000	0,000	0,000	0,000	0,000	0,000	0,000	0,000	0,000	0,000	0,000	0,000	0,000	0,000	0,000
CEACAM1	rs41887902	66	A66S	tolerated(0.97)	0,222	non-private SAP	0,063	0,000	0,063	0,327	0,000	0,238	0,200	0,220	0,115	0,250	0,292	0,250	0,214	0,111	0,000	0,125	0,308	0,158	0,188	0,071	0,188	0,100	0,250	0,625	0,111	0,300	0,450	0,250	0,357	0,000	0,500	0,000	1,000	0,000
CEACAM1	rs1115476612	71	E71D	tolerated(0.09)	0,055	non-private SAP	0,000	0,000	0,000	0,069	0,333	0,000	0,000	0,000	0,000	0,000	0,000	0,000	0,000	0,000	0,000	0,000	0,000	0,000	0,000	0,167	0,286	0,214	0,353	0,333	0,333	0,000	0,333	0,250	0,125	0,000	0,111	0,000	0,000	0,000
CEACAM1	rs1118147252	72	R72K	tolerated(0.93)	0,062	non-private SAP	0,000	0,000	0,000	0,000	0,000	0,000	0,000	0,000	0,000	0,000	0,000	0,000	0,000	0,000	0,000	0,000	0,000	0,000	0,000	0,389	0,188	0,429	0,206	0,056	0,333	0,583	0,222	0,000	0,313	0,000	0,278	0,000	0,000	0,000
CEACAM1	rs134920364	80	A80V	tolerated(1)	0,008	non-private SAP	0,000	0,000	0,000	0,000	0,000	0,000	0,000	0,004	0,000	0,000	0,000	0,000	0,000	0,000	0,000	0,000	0,000	0,000	0,000	0,000	0,000	0,000	0,000	0,050	0,000	0,000	0,083	0,125	0,000	0,000	0,056	0,000	0,167	0,000
CEACAM1	rs446945633	88	A88S	tolerated(0.67)	0,094	non-private SAP	0,000	0,000	0,000	0,018	0,333	0,000	0,000	0,004	0,000	0,000	0,000	0,000	0,000	0,000	0,000	0,000	0,000	0,000	0,000	0,313	0,500	0,625	0,382	0,350	0,643	0,417	0,375	0,333	0,417	0,000	0,167	0,000	0,500	0,000
CEACAM1	-	88	A88V	tolerated(0.57)	0,094	non-private SAP	0,000	0,000	0,000	0,018	0,333	0,000	0,000	0,004	0,000	0,000	0,000	0,000	0,000	0,000	0,000	0,000	0,000	0,000	0,000	0,313	0,500	0,625	0,382	0,350	0,643	0,417	0,375	0,333	0,417	0,000	0,167	0,000	0,500	0,000
CEACAM1	rs434496181	90	T90I	tolerated(0.43)	0,024	non-private SAP	0,000	0,000	0,125	0,000	0,000	0,000	0,016	0,035	0,036	0,000	0,000	0,050	0,063	0,000	0,286	0,125	0,077	0,000	0,063	0,000	0,000	0,000	0,000	0,000	0,000	0,000	0,000	0,000	0,000	0,000	0,000	0,500	0,000	0,000
CEACAM1	rs800603692	93	P93L	tolerated(0.07)	0,044	non-private SAP	0,000	0,000	0,000	0,000	0,000	0,000	0,000	0,000	0,000	0,000	0,000	0,000	0,000	0,000	0,000	0,000	0,000	0,000	0,000	0,278	0,188	0,375	0,088	0,050	0,250	0,500	0,167	0,250	0,143	0,000	0,188	0,000	0,000	0,000
CEACAM1	-	94	A94T	deleterious(0.03)	0,006	non-private SAP	0,000	0,000	0,000	0,000	0,000	0,000	0,000	0,013	0,000	0,000	0,000	0,000	0,000	0,000	0,000	0,000	0,000	0,000	0,000	0,000	0,000	0,000	0,000	0,000	0,000	0,000	0,000	0,000	0,000	0,000	0,000	0,000	0,500	0,000
CEACAM1	rs468450554	100	T100A	deleterious(0.02)	0,177	non-private SAP	0,148	0,500	0,313	0,161	0,000	0,261	0,094	0,175	0,286	0,333	0,167	0,300	0,063	0,214	0,429	0,375	0,346	0,074	0,188	0,167	0,056	0,125	0,056	0,100	0,167	0,150	0,200	0,250	0,167	0,333	0,167	0,500	0,500	0,000
CEACAM1	rs41887905	101	L101F	deleterious(0.01)	0,403	non-private SAP	0,482	0,500	0,563	0,370	0,500	0,478	0,484	0,552	0,500	0,250	0,542	0,550	0,500	0,417	0,500	0,625	0,462	0,563	0,625	0,167	0,050	0,188	0,079	0,000	0,111	0,050	0,000	0,125	0,056	0,500	0,056	0,500	0,000	1,000
CEACAM1	-	111	S111N	tolerated(1)	0,571	non-private SAP	0,714	1,000	0,625	0,536	0,500	0,625	0,578	0,623	0,607	0,417	0,542	0,650	0,500	0,500	0,643	0,688	0,500	0,703	0,813	0,389	0,389	0,625	0,389	0,450	0,500	0,350	0,409	0,375	0,450	0,833	0,278	1,000	0,167	0,500
CEACAM1	-	115	K115E	tolerated(0.66)	0,002	non-private SAP	0,000	0,000	0,000	0,000	0,000	0,000	0,000	0,000	0,000	0,000	0,000	0,000	0,000	0,000	0,000	0,000	0,000	0,000	0,000	0,000	0,000	0,063	0,000	0,000	0,000	0,000	0,000	0,125	0,000	0,000	0,000	0,000	0,000	0,000
CEACAM1	-	121	M121L	deleterious(0.03)	0,079	non-private SAP	0,074	0,000	0,063	0,103	0,000	0,130	0,030	0,089	0,000	0,250	0,167	0,100	0,125	0,000	0,357	0,125	0,385	0,053	0,188	0,063	0,000	0,000	0,000	0,000	0,000	0,000	0,000	0,000	0,000	0,000	0,000	0,000	0,500	0,000
CEACAM1	rs384718213	121	M121T	tolerated(1)	0,500	non-private SAP	0,537	0,500	0,625	0,466	1,000	0,565	0,547	0,586	0,607	0,500	0,542	0,550	0,563	0,600	0,500	0,625	0,500	0,592	0,625	0,313	0,333	0,375	0,306	0,300	0,500	0,100	0,273	0,250	0,150	0,500	0,167	0,500	0,000	1,000
CEACAM1	rs381936589	121	M121I	deleterious(0.04)	0,509	non-private SAP	0,537	0,500	0,625	0,466	1,000	0,587	0,547	0,585	0,607	0,500	0,583	0,650	0,563	0,600	0,500	0,625	0,538	0,592	0,688	0,313	0,333	0,375	0,306	0,300	0,500	0,100	0,273	0,250	0,150	0,500	0,167	0,500	0,500	0,500
CEACAM1	-	124	V124I	tolerated(1)	0,080	non-private SAP	0,074	0,000	0,063	0,121	0,000	0,125	0,031	0,089	0,036	0,250	0,167	0,100	0,125	0,000	0,286	0,125	0,385	0,064	0,125	0,000	0,000	0,000	0,000	0,000	0,000	0,000	0,000	0,000	0,000	0,000	0,056	0,000	0,500	0,000
CEACAM1	-	134	Q134P	tolerated(0.09)	0,270	non-private SAP	0,071	0,000	0,063	0,345	0,000	0,188	0,303	0,235	0,179	0,250	0,292	0,250	0,313	0,200	0,000	0,125	0,269	0,203	0,125	0,100	0,444	0,214	0,528	0,500	0,500	0,500	0,550	0,500	0,556	0,000	0,625	0,000	0,500	0,000
CEACAM1	rs455967697	137	H137Q	tolerated(1)	0,032	non-private SAP	0,054	0,000	0,125	0,034	0,000	0,000	0,047	0,026	0,071	0,000	0,042	0,100	0,063	0,000	0,143	0,000	0,038	0,013	0,000	0,000	0,000	0,000	0,059	0,000	0,063	0,000	0,045	0,000	0,000	0,000	0,000	0,500	0,167	0,000
CEACAM1	rs41887907	140	I140V	tolerated(1)	0,364	non-private SAP	0,161	0,000	0,250	0,426	0,167	0,283	0,391	0,306	0,250	0,417	0,364	0,300	0,438	0,350	0,143	0,188	0,500	0,205	0,188	0,000	0,563	0,417	0,529	0,667	0,500	0,917	0,700	0,625	0,625	0,167	0,875	0,000	1,000	0,000
CEACAM1	rs458061681	141	L141H	tolerated(0.31)	0,017	non-private SAP	0,018	0,000	0,063	0,000	0,000	0,000	0,000	0,022	0,036	0,000	0,091	0,100	0,071	0,000	0,071	0,000	0,000	0,013	0,000	0,000	0,000	0,000	0,000	0,000	0,000	0,000	0,045	0,000	0,000	0,000	0,000	0,000	0,167	0,000
CEACAM1	-	142	P142T	tolerated(0.34)	0,001	Private SAP	0,000	0,000	0,000	0,000	0,000	0,000	0,000	0,000	0,000	0,000	0,000	0,000	0,000	0,050	0,000	0,000	0,000	0,000	0,000	0,000	0,000	0,000	0,000	0,000	0,000	0,000	0,000	0,000	0,000	0,000	0,000	0,000	0,000	0,000
CEACAM1	rs41882877	142	P142Q	tolerated(0.21)	0,276	non-private SAP	0,111	0,000	0,063	0,462	0,500	0,283	0,371	0,267	0,286	0,333	0,333	0,350	0,250	0,350	0,071	0,188	0,308	0,289	0,188	0,278	0,250	0,125	0,289	0,550	0,167	0,150	0,227	0,500	0,400	0,000	0,389	0,000	0,000	0,000
CEACAM1	rs41882878	143	M143V	tolerated(0.53)	0,285	non-private SAP	0,111	0,000	0,063	0,462	0,500	0,283	0,371	0,267	0,286	0,333	0,333	0,350	0,250	0,350	0,071	0,188	0,308	0,282	0,188	0,278	0,250	0,125	0,289	0,550	0,167	0,150	0,227	0,500	0,400	0,000	0,389	0,000	1,000	1,000
CEACAM1	rs41882879	148	V148I	tolerated(0.38)	0,278	non-private SAP	0,135	0,000	0,063	0,462	0,500	0,283	0,391	0,263	0,286	0,333	0,364	0,350	0,250	0,350	0,071	0,188	0,308	0,300	0,188	0,313	0,250	0,125	0,289	0,500	0,167	0,167	0,273	0,500	0,400	0,000	0,389	0,000	0,000	0,000
CEACAM1	rs41882880	181	N181S	tolerated(0.07)	0,283	non-private SAP	0,167	0,000	0,063	0,429	0,500	0,304	0,371	0,258	0,286	0,417	0,292	0,350	0,313	0,500	0,071	0,188	0,308	0,286	0,188	0,278	0,350	0,125	0,263	0,450	0,167	0,300	0,300	0,500	0,400	0,000	0,389	0,000	0,000	1,000
CEACAM1	rs382903321	188	S188G	tolerated(0.12)	0,184	non-private SAP	0,296	0,000	0,375	0,196	0,000	0,261	0,063	0,191	0,464	0,250	0,125	0,500	0,188	0,150	0,571	0,375	0,000	0,300	0,563	0,000	0,000	0,000	0,000	0,000	0,056	0,000	0,000	0,000	0,000	1,000	0,000	1,000	0,000	0,000
CEACAM1	-	223	V223A	tolerated(1)	0,003	non-private SAP	0,000	0,000	0,000	0,000	0,000	0,000	0,000	0,000	0,000	0,000	0,000	0,000	0,000	0,000	0,000	0,000	0,000	0,000	0,000	0,000	0,000	0,000	0,026	0,000	0,000	0,000	0,000	0,125	0,050	0,000	0,000	0,000	0,000	0,000
CEACAM1	rs440339597	233	V233L	tolerated(0.2)	0,001	Private SAP	0,000	0,000	0,000	0,000	0,000	0,000	0,000	0,000	0,000	0,000	0,000	0,000	0,000	0,000	0,000	0,000	0,000	0,000	0,000	0,000	0,000	0,000	0,000	0,000	0,000	0,000	0,000	0,000	0,050	0,000	0,000	0,000	0,000	0,000
CEACAM1	-	235	Y235H	tolerated(0.08)	0,001	Private SAP	0,000	0,000	0,000	0,000	0,000	0,000	0,000	0,000	0,000	0,000	0,000	0,000	0,000	0,000	0,000	0,000	0,000	0,000	0,000	0,000	0,000	0,000	0,000	0,000	0,000	0,000	0,000	0,000	0,050	0,000	0,000	0,000	0,000	0,000
CEACAM1	rs524246474	276	R276Q	tolerated(0.82)	0,050	non-private SAP	0,000	0,000	0,000	0,054	0,333	0,023	0,000	0,000	0,000	0,000	0,000	0,000	0,000	0,000	0,000	0,000	0,000	0,000	0,000	0,167	0,150	0,250	0,237	0,444	0,333	0,050	0,250	0,250	0,100	0,000	0,056	0,000	0,000	0,000
CEACAM1	rs207899037	328	Y328N	tolerated(0.26)	0,098	non-private SAP	0,000	0,000	0,000	0,000	0,000	0,000	0,000	0,000	0,000	0,000	0,000	0,000	0,000	0,000	0,000	0,000	0,000	0,000	0,000	0,500	0,400	0,438	0,444	0,278	0,444	0,700	0,500	0,250	0,450	0,000	0,556	0,000	0,000	0,000
CEACAM1	-	48-49	GS/A	-	0,001	Private SAP	0,000	0,000	0,000	0,000	0,000	0,000	0,000	0,000	0,000	0,000	0,000	0,000	0,000	0,050	0,000	0,000	0,000	0,000	0,000	0,000	0,000	0,000	0,000	0,000	0,000	0,000	0,000	0,000	0,000	0,000	0,000	0,000	0,000	0,000
CEACAM1	-	85-87	D85_N87_del	-	0,461	non-private SAP	0,625	1,000	0,563	0,389	0,500	0,522	0,516	0,583	0,577	0,500	0,625	0,600	0,438	0,563	0,500	0,688	0,462	0,662	0,688	0,278	0,071	0,313	0,139	0,050	0,143	0,071	0,071	0,167	0,083	0,833	0,111	0,500	0,000	0,000
DPP4	-	87	I87V	tolerated(1)	0,001	Private SAP	0,000	0,000	0,000	0,000	0,000	0,000	0,000	0,000	0,000	0,000	0,000	0,000	0,000	0,000	0,000	0,000	0,000	0,000	0,000	0,000	0,000	0,000	0,000	0,000	0,056	0,000	0,000	0,000	0,000	0,000	0,000	0,000	0,000	0,000
DPP4	rs1117179225	137	N137K	tolerated(0.53)	0,004	Private SAP	0,000	0,000	0,000	0,000	0,000	0,000	0,000	0,000	0,000	0,000	0,000	0,000	0,000	0,000	0,000	0,000	0,000	0,000	0,000	0,000	0,000	0,000	0,000	0,000	0,000	0,000	0,000	0,000	0,000	0,667	0,000	0,000	0,000	0,000
DPP4	rs378991725	199	D199N	deleterious(0)	0,018	non-private SAP	0,000	0,000	0,000	0,000	0,000	0,040	0,000	0,000	0,000	0,000	0,000	0,000	0,250	0,063	0,000	0,125	0,000	0,097	0,125	0,000	0,000	0,000	0,000	0,000	0,000	0,000	0,000	0,000	0,000	0,000	0,000	0,000	0,000	0,000
DPP4	-	233	P233S	tolerated(0.09)	0,002	Private SAP	0,000	0,000	0,000	0,000	0,000	0,000	0,000	0,000	0,000	0,000	0,000	0,000	0,000	0,000	0,000	0,000	0,000	0,000	0,000	0,000	0,000	0,000	0,000	0,000	0,000	0,000	0,000	0,000	0,100	0,000	0,000	0,000	0,000	0,000
DPP4	-	251	V251M	tolerated(0.19)	0,001	Private SAP	0,000	0,000	0,000	0,000	0,000	0,000	0,000	0,000	0,000	0,000	0,000	0,000	0,000	0,000	0,000	0,000	0,038	0,000	0,000	0,000	0,000	0,000	0,000	0,000	0,000	0,000	0,000	0,000	0,000	0,000	0,000	0,000	0,000	0,000
DPP4	rs719669768	277	P277L	deleterious(0.04)	0,012	non-private SAP	0,000	0,000	0,000	0,000	0,000	0,000	0,000	0,000	0,000	0,000	0,000	0,000	0,000	0,000	0,000	0,000	0,000	0,000	0,000	0,056	0,050	0,063	0,079	0,100	0,111	0,000	0,000	0,250	0,000	0,000	0,000	0,000	0,000	0,000
DPP4	-	332	S332F	tolerated(0.06)	0,010	Private SAP	0,000	0,000	0,000	0,000	0,000	0,000	0,000	0,000	0,000	0,000	0,000	0,000	0,000	0,000	0,000	0,000	0,385	0,000	0,000	0,000	0,000	0,000	0,000	0,000	0,000	0,000	0,000	0,000	0,000	0,000	0,000	0,000	0,000	0,000
DPP4	rs471811811	353	V353G	deleterious(0)	0,001	Private SAP	0,000	0,000	0,000	0,000	0,000	0,000	0,000	0,004	0,000	0,000	0,000	0,000	0,000	0,000	0,000	0,000	0,000	0,000	0,000	0,000	0,000	0,000	0,000	0,000	0,000	0,000	0,000	0,000	0,000	0,000	0,000	0,000	0,000	0,000
DPP4	-	489	E/AYCRALVADSNFV	-	0,001	Private SAP	0,000	0,000	0,000	0,000	0,000	0,000	0,000	0,004	0,000	0,000	0,000	0,000	0,000	0,000	0,000	0,000	0,000	0,000	0,000	0,000	0,000	0,000	0,000	0,000	0,000	0,000	0,000	0,000	0,000	0,000	0,000	0,000	0,000	0,000
DPP4	-	620	D620N	tolerated(0.17)	0,001	Private SAP	0,000	0,000	0,000	0,000	0,000	0,000	0,000	0,000	0,000	0,000	0,000	0,000	0,000	0,000	0,000	0,000	0,000	0,000	0,000	0,000	0,000	0,000	0,000	0,000	0,000	0,000	0,000	0,000	0,050	0,000	0,000	0,000	0,000	0,000
DPP4	rs714111111	625	I625V	deleterious(0.01)	0,011	non-private SAP	0,000	0,000	0,000	0,000	0,000	0,000	0,000	0,000	0,000	0,000	0,000	0,000	0,000	0,000	0,000	0,000	0,000	0,000	0,000	0,056	0,050	0,125	0,079	0,056	0,125	0,000	0,000	0,125	0,000	0,000	0,000	0,000	0,000	0,000
DPP4	-	634	V634I	deleterious(0.03)	0,001	Private SAP	0,000	0,000	0,000	0,000	0,000	0,000	0,000	0,000	0,000	0,000	0,042	0,000	0,000	0,000	0,000	0,000	0,000	0,000	0,000	0,000	0,000	0,000	0,000	0,000	0,000	0,000	0,000	0,000	0,000	0,000	0,000	0,000	0,000	0,000
FURIN	-	8	F8L	tolerated(1)	0,001	Private SAP	0,000	0,000	0,000	0,000	0,000	0,000	0,000	0,000	0,000	0,000	0,000	0,000	0,000	0,000	0,000	0,000	0,000	0,000	0,000	0,000	0,000	0,000	0,000	0,000	0,000	0,000	0,000	0,000	0,000	0,000	0,071	0,000	0,000	0,000
FURIN	-	8	F8L	tolerated(1)	0,001	Private SAP	0,000	0,000	0,000	0,000	0,000	0,000	0,000	0,000	0,000	0,000	0,000	0,000	0,000	0,000	0,000	0,000	0,000	0,000	0,000	0,000	0,000	0,000	0,000	0,000	0,000	0,000	0,000	0,000	0,000	0,000	0,071	0,000	0,000	0,000
FURIN	-	14	A14T	tolerated(0.47)	0,001	Private SAP	0,000	0,000	0,000	0,000	0,000	0,000	0,000	0,000	0,000	0,000	0,000	0,000	0,000	0,000	0,000	0,000	0,000	0,000	0,000	0,000	0,000	0,000	0,000	0,000	0,000	0,000	0,000	0,000	0,000	0,000	0,071	0,000	0,000	0,000
FURIN	rs439869249	16	A16T	tolerated(0.41)	0,006	non-private SAP	0,042	0,000	0,000	0,000	0,000	0,026	0,000	0,009	0,000	0,000	0,000	0,000	0,000	0,000	0,000	0,000	0,000	0,000	0,000	0,000	0,000	0,000	0,000	0,000	0,000	0,000	0,000	0,000	0,000	0,000	0,071	0,000	0,000	0,000
FURIN	-	21	V21A	tolerated(0.79)	0,001	Private SAP	0,000	0,000	0,000	0,000	0,000	0,000	0,000	0,000	0,000	0,000	0,000	0,000	0,000	0,000	0,000	0,000	0,000	0,000	0,000	0,000	0,000	0,000	0,000	0,000	0,000	0,000	0,000	0,000	0,000	0,000	0,083	0,000	0,000	0,000
FURIN	-	27	E/GPX	-	0,001	Private SAP	0,000	0,000	0,000	0,000	0,000	0,000	0,000	0,000	0,000	0,000	0,000	0,000	0,000	0,000	0,000	0,000	0,000	0,000	0,000	0,000	0,000	0,000	0,000	0,000	0,000	0,000	0,000	0,000	0,000	0,000	0,083	0,000	0,000	0,000
FURIN	-	37	H37R	tolerated(1)	0,002	non-private SAP	0,000	0,000	0,000	0,000	0,000	0,000	0,000	0,000	0,000	0,000	0,000	0,000	0,000	0,000	0,000	0,000	0,000	0,000	0,000	0,000	0,000	0,000	0,028	0,000	0,000	0,000	0,000	0,000	0,000	0,000	0,071	0,000	0,000	0,000
FURIN	rs1116096489	44	V44I	tolerated(0.09)	0,022	non-private SAP	0,000	0,000	0,000	0,000	0,000	0,000	0,000	0,000	0,000	0,000	0,000	0,000	0,000	0,000	0,000	0,000	0,000	0,000	0,000	0,063	0,188	0,071	0,289	0,000	0,000	0,000	0,050	0,000	0,000	0,500	0,000	0,000	0,000	0,000
FURIN	-	46	D46N	tolerated(0.19)	0,001	Private SAP	0,000	0,000	0,000	0,000	0,000	0,000	0,000	0,000	0,000	0,000	0,000	0,000	0,000	0,000	0,000	0,000	0,000	0,000	0,000	0,000	0,000	0,000	0,028	0,000	0,000	0,000	0,000	0,000	0,000	0,000	0,000	0,000	0,000	0,000
FURIN	-	47	R47S	tolerated(0.05)	0,001	Private SAP	0,000	0,000	0,000	0,000	0,000	0,000	0,000	0,000	0,000	0,000	0,000	0,000	0,000	0,000	0,000	0,000	0,000	0,000	0,000	0,000	0,000	0,000	0,028	0,000	0,000	0,000	0,000	0,000	0,000	0,000	0,000	0,000	0,000	0,000
FURIN	-	105	A105T	tolerated(0.76)	0,002	non-private SAP	0,000	0,000	0,000	0,000	0,000	0,000	0,000	0,000	0,000	0,000	0,000	0,000	0,000	0,000	0,000	0,000	0,000	0,000	0,000	0,000	0,000	0,000	0,029	0,000	0,000	0,000	0,000	0,000	0,000	0,000	0,083	0,000	0,000	0,000
FURIN	-	109	I109M	deleterious(0.03)	0,002	non-private SAP	0,000	0,000	0,000	0,000	0,000	0,000	0,000	0,000	0,000	0,000	0,000	0,000	0,000	0,000	0,000	0,000	0,000	0,000	0,000	0,000	0,000	0,000	0,028	0,000	0,000	0,000	0,000	0,000	0,000	0,000	0,083	0,000	0,000	0,000
FURIN	-	109	I109V	tolerated(1)	0,002	non-private SAP	0,000	0,000	0,000	0,000	0,000	0,000	0,000	0,000	0,000	0,000	0,000	0,000	0,000	0,000	0,000	0,000	0,000	0,000	0,000	0,000	0,000	0,000	0,028	0,000	0,000	0,000	0,000	0,000	0,000	0,000	0,083	0,000	0,000	0,000
FURIN	rs434499037	145	R145H	deleterious(0.03)	0,003	non-private SAP	0,000	0,000	0,000	0,000	0,000	0,000	0,000	0,000	0,000	0,000	0,000	0,000	0,000	0,000	0,000	0,000	0,000	0,000	0,000	0,000	0,000	0,071	0,029	0,000	0,000	0,000	0,000	0,000	0,000	0,000	0,083	0,000	0,000	0,000
FURIN	-	399	R399Q	tolerated(0.18)	0,003	non-private SAP	0,000	0,000	0,000	0,000	0,000	0,000	0,000	0,000	0,000	0,000	0,000	0,000	0,000	0,000	0,000	0,000	0,000	0,000	0,000	0,000	0,000	0,000	0,026	0,000	0,056	0,000	0,000	0,000	0,000	0,000	0,063	0,000	0,000	0,000
FURIN	-	575	P575S	tolerated(0.58)	0,001	Private SAP	0,000	0,000	0,000	0,000	0,000	0,000	0,000	0,000	0,000	0,000	0,000	0,000	0,063	0,000	0,000	0,000	0,000	0,000	0,000	0,000	0,000	0,000	0,000	0,000	0,000	0,000	0,000	0,000	0,000	0,000	0,000	0,000	0,000	0,000
FURIN	-	635	I635T	tolerated(0.81)	0,001	Private SAP	0,000	NA	0,000	0,000	0,000	0,000	0,000	0,000	0,000	0,000	0,000	0,000	0,000	0,000	0,000	0,000	0,000	0,000	0,000	0,000	0,000	0,000	0,028	0,000	0,000	0,000	0,000	0,000	0,000	0,000	0,000	0,000	0,000	0,000
FURIN	-	642	T642A	tolerated(0.87)	0,001	Private SAP	0,000	NA	0,000	0,000	0,000	0,000	0,000	0,000	0,000	0,000	0,000	0,000	0,000	0,000	0,000	0,000	0,000	0,000	0,000	0,000	0,000	0,000	0,028	0,000	0,000	0,000	0,000	0,000	0,000	0,000	0,000	0,000	0,000	0,000
FURIN	rs464013150	646	A646T	tolerated(0.06)	0,023	non-private SAP	0,000	NA	0,000	0,000	0,000	0,000	0,000	0,000	0,000	0,000	0,000	0,000	0,000	0,000	0,000	0,000	0,000	0,000	0,000	0,143	0,111	0,167	0,028	0,063	0,071	0,167	0,150	0,000	0,250	0,000	0,063	0,000	0,000	0,000
FURIN	-	656	P656L	tolerated(0.19)	0,002	Private SAP	0,000	0,000	0,000	0,000	0,000	0,000	0,000	0,000	0,000	0,000	0,000	0,000	0,000	0,000	0,000	0,000	0,000	0,000	0,000	0,000	0,000	0,000	0,071	0,000	0,000	0,000	0,000	0,000	0,000	0,000	0,000	0,000	0,000	0,000
FURIN	rs798958151	746	S746R	tolerated_low_confidence(0.44)	0,002	Private SAP	0,000	NA	0,000	0,000	0,000	0,000	0,000	0,000	0,000	0,000	0,000	0,100	0,000	0,000	0,000	0,000	0,000	0,000	0,000	0,000	0,000	0,000	0,000	0,000	0,000	0,000	0,000	0,000	0,000	0,000	0,000	0,000	0,000	0,000
FURIN	-	25-26	RG/X	-	0,001	Private SAP	0,000	0,000	0,000	0,000	0,000	0,000	0,000	0,000	0,000	0,000	0,000	0,000	0,000	0,000	0,000	0,000	0,000	0,000	0,000	0,000	0,000	0,000	0,000	0,000	0,000	0,000	0,000	0,000	0,000	0,000	0,083	0,000	0,000	0,000
TMPRSS2	rs438281619	5	S5P	deleterious_low_confidence(0.04)	0,013	non-private SAP	0,000	0,000	0,000	0,000	0,000	0,043	0,000	0,030	0,000	0,000	0,042	0,000	0,000	0,000	0,143	0,000	0,038	0,000	0,000	0,000	0,000	0,000	0,000	0,000	0,000	0,000	0,000	0,000	0,000	0,000	0,000	0,000	0,000	0,000
TMPRSS2	rs718319764	57	Q57L	tolerated(0.43)	0,013	non-private SAP	0,000	0,000	0,000	0,000	0,000	0,000	0,000	0,000	0,000	0,000	0,000	0,000	0,000	0,000	0,000	0,000	0,000	0,000	0,000	0,000	0,111	0,000	0,026	0,050	0,000	0,125	0,045	0,125	0,100	0,000	0,167	0,000	0,000	0,000
TMPRSS2	-	100	L100F	tolerated(0.07)	0,001	Private SAP	0,000	0,000	0,000	0,000	0,000	0,000	0,000	0,000	0,000	0,000	0,000	0,000	0,000	0,000	0,000	0,000	0,000	0,000	0,000	0,000	0,000	0,000	0,000	0,000	0,056	0,000	0,000	0,000	0,000	0,000	0,000	0,000	0,000	0,000
TMPRSS2	rs452496986	228	V228A	tolerated(0.56)	0,050	non-private SAP	0,000	NA	0,000	0,000	0,000	0,000	0,000	0,000	0,000	0,000	0,000	0,000	0,000	0,000	0,071	0,000	0,000	0,000	0,000	0,167	0,450	0,250	0,158	0,250	0,222	0,300	0,227	0,125	0,150	0,000	0,167	0,000	0,000	0,000
TMPRSS2	rs1116568954	298	E298V	tolerated(0.11)	0,005	non-private SAP	0,000	0,000	0,000	0,000	0,000	0,000	0,000	0,000	0,000	0,000	0,000	0,000	0,000	0,000	0,000	0,000	0,000	0,000	0,000	0,000	0,063	0,000	0,000	0,000	0,071	0,188	0,000	0,000	0,000	0,000	0,000	0,000	0,000	0,000
TMPRSS2	rs136400585	421	A421T	tolerated(1)	0,236	non-private SAP	0,074	0,000	0,143	0,315	0,000	0,239	0,241	0,145	0,769	0,300	0,375	0,050	0,375	0,111	0,143	0,375	0,115	0,212	0,000	0,722	0,250	0,438	0,553	0,444	0,222	0,350	0,333	0,125	0,278	0,167	0,250	0,000	0,000	0,000
TMPRSS2	rs209763791	429	R429Q	tolerated(1)	0,228	non-private SAP	0,071	0,000	0,000	0,310	0,000	0,217	0,226	0,149	0,714	0,333	0,292	0,050	0,375	0,100	0,143	0,188	0,077	0,162	0,000	0,722	0,222	0,438	0,553	0,500	0,222	0,333	0,400	0,250	0,250	0,167	0,250	0,000	0,000	0,000

Table S4. ACE2 residues critical for protein function and coronavirus pathogenesis.

Interaction sites[1]

HSA*	Q24	A25	K26	T27	F28	D30	K31	H34	E35	E37	D38	Y41	Q42	L45	K68	L79	M82	Y83	N90	Q325	E329	N330	V339	K353	G354	D355	R357	M383	P389	R393	S425	P426	D427	R559
BTA^	na	na	na	na	na	na	na	na	na	na	na	na	na	na	K7	M18	T21	Y22	N29	Q263	D267	N268	G277	K291	G292	D293	R295	M321	P327	R331	A363	P364	D365	R497
NGS§	.	.	.	.	.	.	.	.	.	.	.	.	.	.	.	**I18**	.		.	.	.	.	.	.	.	.	.	.	.	.	.	.	.	.

Binding/Active sites[2]

	Binding site	Binding site	Binding site	Binding site	Metal binding	Active site	Metal binding	Metal binding	Binding site	Binding site	Binding site	Binding site
HSA	R273	H345	P346	T371	H374	E375	H378	E402	W477	K481	H505	Y515
BTA	R211	H283	P284	T309	H312	E313	H316	E340	W415	K419	H443	Y453
NGS	.	.	.	.	.	.	.	.	.	.	.	.

Cleavage by ADAM17[2]

HSA	Q653	Y654	F655	L656	K657	V658	K659
BTA	K59	Y59	F59	S59	E59	A59	R5
NGS	Q591	.	.	.	A595	.	K597

Cleavage by TMPRSS1 and TMPRSS2[2]

HSA	T698	E699	V700	E701	K702	A703	I704	R705	M706	S707	R708	S709	R710	I711	N712	D713	A714	F715	R716
BTA	T636	E637	V638	E639	N640	A641	I642	R643	L644	S645	R646	D647	R648	I649	N650	D651	V652	F653	Q654
NGS	.	.	.	.	.	.	.	.	.	.	.	.	.	.	.	.	.	.	.

Glycosylation sites[3]

HSA	N90	N103	N322	N432	N546
BTA	N29	S42	Y260	N370	N484
NGS	.	.	.	.	.

* Residues are based on the human UniProtKB entry Q9BYF1. ^ Residues are based on the bovine UniProtKB entry A0A452DJE0. A dot represents an identical residue where residues differing between human and cattle are reported. § Variants identified in the next generation sequencing datasets. Residues are reported. A dot represents an identical residue. [1] UniProtKB annotations and [30–31,56–57 64–68]. [2] UniProtKB annotations and [31] [3] UniProtKB annotations and [69–71].

Table S5. ANPEP residues critical for protein function and coronavirus pathogenesis.

**Interaction sites[1]**										
HSA*	D288	Y289	V290	E291	K292	Q293	A294	S295	D315	L318
BTA^	T285	S286	V287	E288	S289	V290	A291	P292	L312	L315
NGS[§]	·	·	·	·	·	·	·	·	·	·
**Binding/Active sites[2]**										
	Metal binding	Active site	Metal binding	Metal binding	Site					
HSA	H388	E389	H392	E411	Y477					
BTA	H385	E386	H389	E408	Y474					
NGS	·	·	·	·	·					
**Glycosylation sites[3]**										
HSA	N128	N234	N265	N319	N527	N573	N625	N681	N818	
BTA	N125	N231	S262	N316	N524	T571	N624	N680	Q817	
NGS	·	·	·	·	·	·	·	·	·	

**Interaction sites[4]**										
SSC[#]	E731	N736	W737	N783						
BTA	E733	N738	W739	N785						
NGS[§]	·	·	·	·	·	·	·	·	·	·

[*] Residues are based on the human UniProtKB entry P15144. [^] Residues are based on the bovine UniProtKB entry A0A3Q1MB09. Residues differing between human and cattle are underlined. [#] Residues are based on the porcine UniProtKB entry P151445. Residues differing between pig and cattle are underlined. [§] Variants identified in the next generation sequencing datasets. Residues are reported. A dot represents an identical residue. [1] UniProtKB annotations and [72–73]. [2] UniProtKB annotations and and [74–75]. [3] UniProtKB annotations and [73–74]. [4] UniProtKB annotations and [47,76].

Table S6. CEACAM1 residues critical for protein function and coronavirus pathogenesis.

**Interaction sites[1]**																			
MMU[#]	G63	A64	Y68	T73	I75	D76	R81	V83	N85	S86		M88	F90	T91	G92	Q93	E127	N128	R130
BTA^	L63	G64	Y68	V73	N75	T76	S81	R83	D85	T86		A88	T90	K91	G92	P93	D127	D128	Q130
NGS[§]	.	A64	.	.	.	.	.	.	.	Deletion	S88	V88	I90	.	.	L93	.	.	.

**Glycosylation sites[2]**																			
HSA*	N111	N115	N152	N182	N197	N208	N224	N232	N254	N274	N288	N292	N302	N309	N345	N351	N363	N378	N405
BTA	S111	K115	N152	N182	K197	N208	S224	N232	na	na	na	na	na	na	V257	D263	G275	N290	S317
NGS	N111	E115	.	.	.	.	.	.	.	.	.	.	.	.	.	.	.	.	

* Residues are based on the human UniProtKB entry P13688. ^ Residues are based on the bovine UniProtKB entry A0A3Q1MQ27. A dot represents an identical residue whereas residues differing between human and cattle are reported. [#] Residues are based on the murine UniProtKB entry P97449. A dot represents an identical residue whereas residues differing between mouse and cattle are reported. [§] Variants identified in the next generation sequencing datasets. Residues are reported. A dot represents an identical residue. [1] [25]. [2] UniProtKB annotations and [77–78].

Table S7. DPP4 residues critical for protein function and coronavirus pathogenesis.

**Interaction sites[1]**															
HSA*	K267	F269	Q286	T288	A289	A291	L294	I295	H298	R317	Y322	R336	Q344	I346	K392
BTA^	K266	F268	Q285	V287	P288	G290	L293	I294	H297	R316	Y321	R335	Q343	I345	K391
NGS§	.	.	.	.	.	.	.	.	.	.	.	.	.	.	.
**Active sites[2]**															
HSA	E205	E206	Y547	S630	D708	H740									
BTA	E204	E205	Y546	S629	D707	H739									
NGS	.	.	.	.	.	.									
**Glycosylation sites[3]**															
HSA	N85	N92	N150	N219	N229	N281	N321	N520	N685						
BTA	N84	N91	N149	N218	N228	N280	N320	H519	N684						
NGS	.	.	.	.	.	.	.	.	.						

* Residues are based on the human UniProtKB entry P27487. ^ Residues are based on the bovine UniProtKB entry P81425. Residues differing between human and cattle are underlined. § Variants identified in the next generation sequencing datasets. Residues are reported. A dot represents an identical residue. [1] UniProtKB annotations and [79–80].. [2] UniProtKB annotations and [81]. [3] UniProtKB annotations and [71,82–85].

Table S8. FURIN residues critical for protein function and coronavirus pathogenesis.

**Cleavage/Binding/Active/ sites**[1]														
	Cleavage	Cleavage	Cleavage	Cleavage	Cleavage	Active site	Substrate	Active site	Substrate	Substrate	Substrate	Active site	Substrate binding	Substrate binding;
HSA*	V72	R75	S76	R107	D108	D153	D154	H194	V231	E236	D264	D306	Y308	S368
BTA^	V72	R75	S76	R107	D108	D153	D154	H194	V231	E236	D264	D306	Y308	S368
NGS§	.	.	.	.	.	.	.	.	.	.	.	.	.	.

**Glycosilation sites**[2]			
	Glycosilation	Glycosilation	Glycosilation
HSA*	N387	N440	N553
BTA^	N387	N440	N553
NGS§	.	.	.

*Residues are based on the human UniProtKB entry P09958. ^ Residues are based on the bovine UniProtKB entry B0JYR0. Residues differing between human and cattle are underlined. § Variants identified in the next generation sequencing datasets. Residues are reported. A dot represents an identical residue. [1] UniProtKB annotations

Table S9. TMPRSS2 residues critical for protein function and coronavirus pathogenesis.

**Cleavage/Binding/Active sites[1]**								
	Cleavage	Cleavage	Active site	Active site	substrate	Active site	substrate	substrate
HSA*	R255	I256	H296	I346	D435	S441	S460	G462
SSC^	R253	I254	H294	I344	D433	S439	S458	G460
NGS§	.	.	.	.	.	.	.	.

* Residues are based on the human UniProtKB entry O15393.

^ Residues are based on the bovine UniProtKB entry A2VDV7. Residues differing between human and cattle are underlined. § Variants identified in the next generation sequencing datasets. Residues are reported. A dot represents an identical residue. [1] [30,32,86].

References

References are reported in the main text.
